# Vermicompost Supply Modifies Chemical Composition and Improves Nutritive and Medicinal Properties of Date Palm Fruits From Saudi Arabia

**DOI:** 10.3389/fpls.2019.00424

**Published:** 2019-04-11

**Authors:** Soad Al Jaouni, Samy Selim, Sherif H. Hassan, Hussein S. H. Mohamad, Mohammed A. M. Wadaan, Wael N. Hozzein, Han Asard, Hamada AbdElgawad

**Affiliations:** ^1^Department of Hematology and Yousef Abdullatif Jameel Chair of Prophetic Medicine Application, Faculty of Medicine, King Abdulaziz University, Jeddah, Saudi Arabia; ^2^Department of Clinical Laboratory Sciences, College of Applied Medical Sciences, Jouf University, Sakakah, Saudi Arabia; ^3^Department of Botany, Faculty of Science, Suez Canal University, Ismailia, Egypt; ^4^Botany and Microbiology Department, Faculty of Science, Beni-Suef University, Beni-Suef, Egypt; ^5^Department of Chemistry, Faculty of Science, Beni-Suef University, Beni-Suef, Egypt; ^6^Bioproducts Research Chair, Department of Zoology, College of Sciences, King Saud University, Riyadh, Saudi Arabia; ^7^Laboratory Integrated Molecular Plant Physiology Research, Department of Biology, University of Antwerp, Antwerp, Belgium

**Keywords:** date palm fruits, bio-fertilizer, vermicomposts, medicinal potential, nutritive quality, metabolic profile, β-D-glucogallin

## Abstract

To meet the increased demand for phytochemicals, plant cultivation in soil amended with biofertilizers has been developed. Here, we aimed to use vermicompost as an environmentally safe biofertilizer to enhance the nutritive and medicinal value of five common cultivars of Saudi date palm; namely *Phoenix dactylifera* L. var. Ajwa, Hulwa, Ruthana, Sefri, and Luban. To determine changes in the fruit nutritive composition, primary metabolites, antioxidants, phenolic compounds and mineral profiles were analyzed in the fruits from non-fertilized and vermicompost-fertilized date palms. We also tested how changes in the fruit chemical compositions due to vermicompost fertilization affected their medicinal potentials. Applying vermicomposts generally increased primary metabolites, vitamins, and mineral content as well as the medicinal potential of the date palm fruits. This positive effect is possibly explained by the role of vermicomposts in improving soil health and fertility. Furthermore, clustering analyses and principal component analysis (PCA) indicated cultivar-specific responses. PCA analysis also revealed that the bioactivities of the date palm fruit extracts and their antioxidants tended to display correlated output values. One of the highly accumulated phenolic compounds, β-D-glucogallin, was extracted and purified from *P. dactylifera* L. var. Ajwa fruits and showed significant antioxidant, anticancer, antibacterial, antimutagenic, and antiprotozoal activities. Overall, applying vermicompost is an innovative approach to increase the nutritive quality and medicinal potential of date palm fruits.

## Introduction

Fruits constitute a major part of the daily human diet, and in the Middle East date fruits are particularly important because of their nutritive value. Date palms (*Phoenix dactylifera* L., Palmae) are the source of these fruits and are widely cultivated in the Middle East (Al-Farsi and Lee, 2008). There are about 5000 varieties of date palm fruits grown in different regions of the world. Date palm cultivation is not only for food, but also supports specific social development of people located in very hot regions such as Saudi Arabia (Selim et al., 2012). For the natives in the Middle East, date palm fruits are used as a staple carbohydrate. Date palm fruits are rich in amino acids, fatty acids (FA), and minerals (Al-Shahib and Marshall, 2003) and contain high levels of phenolic compounds, vitamins and dietary fibers, which increase their nutritive and therapeutic value (Al-Shahib and Marshall, 2003). Extracts of date palm fruits were reported to have antibacterial, antioxidant and antifungal properties (Selim et al., 2012; Hamad et al., 2015). They have the potential to supply antioxidants in different pharmaceutical and medicinal applications. For instance, the extracts reduced cytotoxicity of induced liver cancer, restored liver function, and protected against hepatotoxicity of carbon tetrachloride (Hussain et al., 2014; Khalid et al., 2017). Considering the nutritive values of date palm fruits, the importance of studying their biochemical composition, nutritive, and medicinal potential is increasingly recognized.

From an ecological point of view, there is an increasing demand to apply eco-friendly approaches to improve productivity and food quality of agricultural crops. Applying bio-fertilizers containing beneficial microorganisms increased plant yield and helped in maintaining soil health and fertility (O’Connell, 1992; Nagavallemma et al., 2004). One widely used bio-fertilizer is vermicompost, a mixture of organic materials resulting from interactions between microorganisms and earthworms. This biofertilizer is rich in minerals, plant growth hormones and humic acids in addition to providing beneficial microbial populations (Theunissen et al., 2010). Applying vermicompost for 60 days improved marigold growth and yield (Gupta et al., 2014). Similarly, Gutiérrez-Miceli et al. (2007) determined that sheep-manure vermicompost improved yield and carbohydrate content in tomato fruits (*Lycopersicum esculentum*).

Vermicompost is rich in humic acids and thereby provides a supportive environment for chemical reactions and microbial populations (Nagavallemma et al., 2004). In addition, increased N, P, K, Cu, Mg, and Fe levels were reported in soils fertilized with vermicompost, thus enhancing plant metabolic functions (Tanaka et al., 1998). Plants fertilized with vermicompost showed enhanced root growth and development, which could explain the improved macro- and micro-nutrient uptake and assimilation (Theunissen et al., 2010). On the other hand, the positive effect of vermicompost was dependent on the applied fertilizer level, as high vermicompost levels in the soil reduced soil aeration and salt concentrations (Tucker, 2005) that could inhibit plant growth (Subler et al., 1998).

Recent approaches have been successfully used to investigate primary and secondary metabolites in date palm fruits and to assess their nutritive composition and medicinal potential. In our previous study, high performance liquid chromatography (HPLC) and mass spectrometry (HPLC/PDA/MS) were used to measure primary and secondary metabolites (soluble sugars, amino acids, organic acids, FA, phenolics, and flavonoids) in 12 cultivars of date palm fruits (Hamad et al., 2015). One molecule induced in date palm fruits by the vermicompost fertilization, that could be important in date fruit medicinal properties, is the phenolic compound β-D-glucogallin. This molecule acts as an antioxidant and has antimicrobial activities (Chanwitheesuk et al., 2007; Alu’datt et al., 2017; Rajan and Muraleedharan, 2017). β-D-glucogallin has been extracted for medical applications (Alu’datt et al., 2017) and has been reported to have anti-inflammatory and anti-cancer effects (Puppala et al., 2012).

In this study, we aimed to understand the potential effect of vermicompost fertilization on soil fertility and yield, nutritive quality and the medicinal potential of date palm fruits. Special emphasis was given to the accumulation of health-beneficial phytochemicals (primary metabolites, minerals, vitamins, and antioxidants). Another aim was to evaluate the bioactivity of an isolated phenolic compound, β-D-glucogallin, from *P. dactylifera* cv. Ajwa.

## Materials and Methods

### Experimental Design

The field study was performed during two successive growing seasons (2015 and 2016) in a private orchard located at Sakakah, Al Jawf region in the north of Saudi Arabia (latitude: 13.837832, longitude: 45.834881). Five cultivars of Saudi date palm (Ajwa, Hulwa, Ruthana, Sefri, and Luban) of 12 years of age were planted in sandy soil, in spaces of 6 × 6 m. Selected trees were healthy and as uniform as possible in age and growth. The number of leaves per bunch (9:1 ratio) was adjusted for each control and treated cultivars. No fertilizers were applied before the study. Date palm fruits were collected from the same trees in 2015 and 2016. In March of each year, 10 kg of vermicomposts were applied to the soil surface and then followed by irrigation. The experiment was conducted with three replicates for each treatment and three trees for each replicate; therefore, we used 12 trees for each cultivar. Soil water content was monitored by neutron moisture meter (neutron probe) and maintained at 60% of the soil water capacity. After 6 months, date palm fruits were collected from treated and untreated cultivars at the fully ripe (Tamar) stage. Washed date palm fruits were stored at -40°C before measurement of metabolic and mineral composition and extraction for biological activities evaluation. Results obtained for both harvests (2015 and 2016) were averaged.

### Soil Analysis

Soil physicochemical properties, pH, electrical conductivity (EC), organic matter (OM), total phenolic compounds and mineral nutrients were determined according to Jones (2001).

## Metabolite Determinations

### Sugars

Mono, di- and polysaccharides in the date were measured by using HPLC following Hamad et al. (2015). 150 mg of dry plant tissues were vigorously homogenized in 2 ml of acetonitrile:water (1:1 v/v) for 5 min. Samples were boiled for denaturation of sugar enzymes and after 15 min of incubation at 55–60°C in a water bath, filtration was performed through Whatman No. 1 filter paper. Another 20 ml of solvent was added to the remaining pulp for re-extraction. The mobile phase was a mixture of acetonitrile and water (75:25 v/v) and was applied at a flow rate of 1 ml min^-1^. Sugars were identified and quantified according to a calibration curve (1–10 mg 100 ml^-1^) of the corresponding standards of the same extracting solution.

### Organic Acids

Quantitative determination of organic acids was performed according to Hamad et al. (2015). Briefly, 500 mg of powdered fruits were extracted in phosphoric acid (0.1%). After centrifugation for 30 min at 14,000 *g* and 4°C, the supernatants were passed through Millipore microfilters (0.2 μm pore size). Organic acids were detected by HPLC detection methods coupled with SUPELCOGELC-610H column and UV detector (LaChrom L-7455 diode array, Japan). Phosphoric acid (0.1%, v/v) at a flow rate of 0.45 ml min^-1^ was used as a mobile phase.

### Amino Acids

One hundred mg of the dry fruit samples was homogenized in 5 ml of 80% aqueous ethanol (MagNA Lyser, Roche, Belgium) for 1 min at 5000 rpm (Hamad et al., 2015). After centrifugation for 25 min at 14,000 *g*, the supernatant was resuspended in 5 ml of absolute chloroform. Immediately, the residue was re-extracted with 1 mL deionized water and centrifugation was repeated. The supernatant and pellet were suspended in absolute chloroform and then centrifuged at 8000 *g* for 10 min. The aqueous phase was filtered using Millipore microfilters (0.2 μM pore size). Amino acids were quantified using a Waters Acquity UPLC TQD system (Milford, Worcester County, MA, United States) coupled to a BEH amide column.

### Fatty Acids

Fatty acid quantification was performed by gas chromatography according to Hamad et al. (2015). 200 mg dry fruit samples extracted in 1 mL aqueous methanol (1:1 w/v) at 25°C until discoloration of the tissues occurred. An internal standard (nonadecanoic acid) was added during extraction. GC/MS analysis was carried out on a Hewlett Packard 6890, MSD 5975 mass spectrometer (Hewlett Packard, United States), equipped with a HP-5 MS column (30 m × 0.25 mm × 0.25 mm). Lipids were identified with the NIST 05 database and Golm Metabolome Database^1^.

### Phenolics and Flavonoids

Phenolics and flavonoids were determined by using the method described in Hamad et al. (2015). About 200 mg of the dry date fruits were homogenized in an acetone-water solution (4:1 v/v) for 24 h. Phenolics and flavonoids were quantified on a Shimadzu HPLC system (SCL-10 AVP, Japan), equipped with a Lichrosorb Si-60, 7 μm, 3 × 150 mm column and a diode array detector (SPDM10AVP). The mobile phase water-formic acid (90:10, v/v) and acetonitrile/water/formic acid (85:10:5, v/v/v) was applied at a flow rate of 0.8 mL min^-1^. The extract was filtered, centrifuged and the resulted supernatant was evaporated under vacuum, then the residue was resuspended in methanol (HPLC grade). Baicalein (100 μg/ml) was used as an internal standard, and the concentration of the phenolic compounds was determined based on the corresponding standard.

For determination of the total phenolics and flavonoids, 100 mg of the dry fruit samples was homogenized in 0.5 ml 80% aqueous ethanol. After centrifugation, the pellet was washed twice with 80% ethanol. A Folin–Ciocalteu assay was used for total phenolic content quantification (AbdElgawad et al., 2014), with gallic acid as a standard. Total flavonoid content was estimated according to AbdElgawad et al. (2015) with quercetin as a standard.

### Vitamins

Contents of ascorbate, tocopherols, and carotenoids were determined in date palm fruits by HPLC. The separation and detection processes were done with UV and/or fluorescence detectors (Jakob and Elmadfa, 1996; Siebert, 1999; Van Oostende et al., 2008; AbdElgawad et al., 2015). Tocopherols were extracted in absolute hexane and their separation and quantification were conducted by a normal phase HPLC (Shimadzu, ‘s-Hertogenbosch, Netherlands). Carotenes were extracted in acetone and quantified by a reversed phase HPLC (software analysis with Shimadzu Lab Solutions Lite). Phylloquinone was detected by reversed phase HPLC (RP18 column, Eurosphos-100, 250 × 4.6 mm, Germany) using a fluorescence detector (excitation, 243 nm; emission, 430 nm).

## Minerals

One hundred mg of the dry fruit samples were digested in HNO_3_/H_2_O solution (5:1 v/v) in an oven according to AbdElgawad et al. (2015) and Hamad et al. (2015). Minerals were determined by ICP-MS (Finnigan Element XR, Scientific, Bremen, Germany).

## Biological and Medicinal Activities

### Antibacterial Assay

The disc diffusion method was used to evaluate the bacterial potential of the date palm fruit extracts (Selim et al., 2012). The clinical bacterial strains (*Streptococcus spp*., *Serratia marcescens, Staphylococcus aureus, Escherichia coli, Klebsiella pneumoniae, Proteus vulgaris, Pseudomonas aeruginosa*) obtained from the microbial culture collection of the Department of Clinical Laboratory Sciences, Al Jouf University, Saudi Arabia were used as test strains. About 150 μl of the bacterial suspension was spread on Muller Hinton agar and nutrient agar media. The same solvent employed to extract the date palm fruits was used as a negative control. Amoxicillin (30 μg/disc), gentamycin (30 μg/disc), and streptomycin (30 μg/disc) were used as positive reference standards. The minimal inhibitory concentration (MIC) and minimal bactericidal concentration (MBC) were determined for the strains sensitive to the fruit extract in the assay. Time-kill studies were performed for *S. aureus* in McCartney bottles using a method based on the European Standard quantitative suspension test.

### Anti-mutagenicity Assay (Ames Test)

An Ames assay (Ames et al., 1975) was used to determine the anti-mutagenicity of β-D-glucogallin. The two strains (TA98 and TA100) of *Salmonella typhimurium* were used for this test. Sodium azide (NaN_3_) and mutagen 4-nitro-o-phenylenediamine (NPD) were used as positive controls. Toxicity of β-D-glucogallin was checked against all the used mutagens. The experiments were conducted in pre-incubation and co-incubation modes. The inhibition % of mutagenic activity was calculated as described by Ames et al. (1975), where the number of histidine revertants induced by the mutagen alone, the mutagen in the presence of β-D-glucogallin and β-D-glucogallin alone were used for the calculations.

### Antiprotozoal Activity

The vitro anti-leishmanial assay against the amastigotes of *Leishmania donovani* and *Trypanosoma cruzi* was conducted according to the protocols established by Räz et al. (1997). Amastigote density was 1 × 10^8^ parasites per mL, and 90 μL were added to each well of the 96-well plates. Then, 10 μL of β-D-glucogallin and standard compound (Benznidazole and Miltefosine) solutions were added to the wells. Amastigotes of *L. donovani* were incubated with AlamarBlue^TM^ for 3 days, and the viability was evaluated by a fluorescence scanner. Viability measurements of trypomastigotes of *T. cruzi* and *L. donovani* were conducted using rat skeletal myoblasts and seeded in the microtiter plates using chlorophenol red-β-D-galactopyranoside (CPRG)-Nonidet substrate. After 4 days of incubation at 37°C and under 5% CO_2_ environment, the developed color reaction was read at 540 nm. The IC_50_ values were calculated for both species.

### DPPH Radical Scavenging Activity Assay

The DPPH method was used to measure the free radical scavenging activity of date palm fruit ethanol extracts and β-D-glucogallin (Hamad et al., 2015). The extracts were mixed with 0.25 mM DPPH solution. After shaking for 30 min, 2 mL of distilled water was added, and the absorbance was measured at 517 nm.

### Total Antioxidant Capacity (FRAP)

The total antioxidant capacity (FRAP) was assayed by extracting date palm fruits in 80% ice-cold ethanol in liquid nitrogen. FRAP reagent (2,4,6-tripyridyl-striazine and FeCl_3_ dissolved in 0.3 M Na-acetate buffer) was mixed with the samples or with the standard (trolox) solution in micro-titration plates for 30 min and measured at 593 nm (AbdElgawad et al., 2015).

### Anticancer Activity

Human cancer cell lines [Hepatocellular carcinoma (HepG2), Colon carcinoma (Colo205), Embryonic kidney adenocarcinoma (293), and Urinary bladder carcinoma (T24P)] were used in this study. Ethanol extracts of the tested date palm fruits (2 mg/mL) as well as β-D-glucogallin (0.1 mg/mL) dissolved in ethanol were used. Cell lines were grown in Dulbecco’s Modified Eagle Medium (DMEM) [10% fetal calf serum, Na-pyruvate, streptomycin (1 mg/0.1 mL), penicillin (1 U/0.1 mL)] at 37°C and 5% CO_2_. The cell lines were suspended and seeded in DMEM. Incubation of the cell lines with extracts, β-D-glucogallin and ethanol as a negative control was continued until a confluent growth was achieved. Then, the cells were collected by trypsinization in trypsin solution (0.25% trypsin w/v) and the vital cells were determined by the CellTiter-Blue reagent (Promega, Madison, WI, United States) (Solowey et al., 2014).

### Extraction, Fractionation, and Isolation of β-D-Glucogallin

To isolate β-D-glucogallin, 250 g of *P. dactylifera* cv. Ajwa date fruits, which showed the highest concentration of β-D-glucogallin, was collected and extracted in ethanol for up to 6 h. Then, the filtrate was concentrated under vacuum. Dried ethanol extract was eluted by using gradients of heptane:ethanol (9:1 to 0:1) as a mobile phase to yield seven fractions depending upon thin layer chromatography (TLC) analyses. The abundant fraction, was evaporated under reduced pressure at 45°C to remove the solvent and then subjected to column chromatography (Merck KGaA, Germany) on a silica gel column. The mobile phase (dichloromethane:water) with gradient from 10:0 to 3:10 was chosen for elution depending upon TLC analyses to give 5 sub-fractions (F1-1 to F1-5). Sub-fraction 2 (F5-2) (3.14 g) showed the highest elongation activity by using coleoptiles bioassay. In this assay, 5 mm of wheat coleoptiles were placed in test tubes containing the extracts and controlled tubes were filled with water. Test tubes were moved to growth cabinet for 24 h, and then the increases in collectibles length were recorded. TLC analyses of F1-3 showed the presence of mixture of three compounds, and one of them was very low, the major one was purified by subjecting to Sephadex LH-20 column and 104.5 mg of the purified compound was yielded. The preliminary bioactivity test of the isolated pure compound indicated its phytotoxicity.

### Identification of β-D-Glucogallin Pure Compound

IR, ^1^H, and ^13^C NMR spectra were used to identify the pure isolated phenolic compound β-D-glucogallin. Samples were dissolved in acetone and the internal standard tetramethylsilane (TMS) was used. The compound in F1-3 was identified based on spectroscopic analyses and comparison of ^1^H and ^13^C NMR data with previous literature values (Puppala et al., 2012). A complete attribution was performed on the basis of a 2D experiment (heteronuclear multiple bond correlation, HMBC). The Micromass type QTOF 2 spectrometer was used to measure the resolution-mass spectrometry (HRMS) data.

### Statistical Analysis

One-way analysis of variance (Duncan’s test, *P* ≤ 0.05) was applied by using SPSS 16.0 (SPSS, Woking, Surrey, United Kingdom). PCA analysis was performed by using XLSTAT (software, 2011). All parameters were subjected to Pearson distance metric cluster analysis by using the MultiExperiment Viewer (MeV) TM4 software package (Dana-Farber Cancer Institute, Boston, MA, United States).

## Results and Discussion

Characterization of the nutritive and medicinal potentials of the date palm fruits requires detailed knowledge of the metabolite content and biological activities. Although the nutritive composition of date palm fruits has been reported (Assirey, 2015; Hamad et al., 2015), analyses at multiple-omics levels are combined (Khalid et al., 2017). Moreover, phytochemicals from date palm fruits are believed to possess numerous health benefits for humans and are important in the daily fruit intake for large groups of people. Nevertheless, the generally consumed amount of date palm fruits is less than is optimal for health maintenance. One strategy to improve the intake of these phytochemicals is through increasing the date fruit yield and quality (Khalid et al., 2017). Application of bio-fertilizers such as vermicompost is a low-impact, environmentally friendly and increasingly used technology in agriculture (Domínguez, 2004).

Vermicomposting resulted in a low soil C:N ratio, high water-holding capacity and porosity, in addition to increasing the availability of many nutrients for plants (Theunissen et al., 2010). In contrast to a large number of studies on the effects of regular composting in plants, relatively little is known on the effects of vermicomposting. In this study, we analyzed vermicompost-induced changes in five common varieties of date palm fruits at the level of primary metabolism, vitamins, and minerals, besides the effect on therapeutic properties. Overall, vermicompost improved the nutritive and therapeutic values of date palm fruits growing in both first and second seasons, with no significant impact of season variation on the trend of the measured parameters. Furthermore, we extracted and tested the medicinal activity of one of the most induced phenolic compounds by vermicompost supply.

### Vermicompost Improved Soil Fertility and Date Palm Fruit Yield

Analysis of soil physicochemical properties (Table 1) indicates that vermicompost addition did increase several parameters (minerals and total phenols) but did not affect soil pH and EC. Increased levels of phenolic compounds were observed in the fertilized soil with the highest increase recorded for the Ruthana tree (Table 1). Similar findings have previously been reported by Nagavallemma et al. (2004) and Theunissen et al. (2010), showing significant increases in concentrations of phenolics and amino acids in vermicompost-fertilized soil. With respect to the soil mineral nutrients, vermicompost raised the concentrations of the exchangeable elements, N, Ca, Zn, Mn, Cu, Mg, and P, but did not significantly affect the availability of soil K and Na. Previous investigations in this regard recorded elevated levels of exchangeable N, P, and K in soil incorporated with residues of different plants as compared with unfertilized soil (Theunissen et al., 2010). In addition, some reports pointed out the role of vermicompost in promoting the processes of biological nitrogen fixation and nodulation (Theunissen et al., 2010).

**Table 1:** Vermicompost fertilization effect on the properties of rhizospheric soil and the yield of five palm cultivars.

	Ajwa		Hulwa		Ruthana		Sefri		Luban	
	Control	Treated	Control	Treated	Control	Treated	Control	Treated	Control	Treated
**Soil composition**					
EC dS/m	3.5 ± 0.2^a^	3.6 ± 0.4^a^	3.3 ± 0.2^a^	3.4 ± 0.7^a^	3.2 ± 0.5^a^	3.4 ± 0.5^a^	3.5 ± 0.2^a^	3.7 ± 0.6^b^	3.2 ± 0.9^a^	3.6 ± 0.3^a^
pH	7.8 ± 0.5^a^	7.7 ± 0.4^a^	7.7 ± 0.2^a^	7.6 ± 0.9^a^	7.7 ± 0.6^a^	7.5 ± 0.5^a^	7.6 ± 0.1^a^	7.4 ± 0.5^a^	7.6 ± 0.7^a^	7.5 ± 0.3^a^
HCO3 (meq/L)	9.8 ± 0.7^a^	9.9 ± 0.7^a^	9.5 ± 1.2^a^	10.2 ± 0.7^a^	10.3 ± 0.7^a^	9.8 ± 1.5^b^	9.9 ± 0.3^a^	9.6 ± 1.1^b^	10.5 ± 0.8^a^	10.7 ± 0.2^b^
Ca^++^ (meq/L)	19.2 ± 4.0^a^	24.5 ± 0.5^b^	23.6 ± 0.2^a^	27.6 ± 2.1^a^	18.6 ± 0.2^a^	26.1 ± 1.5^b^	21.6 ± 0.3^a^	28.7 ± 1.3^b^	17.6 ± 0.8^a^	24.3 ± 3.1^b^
Mg^++^ (meq/L)	7.9 ± 0.8^a^	11.2 ± 0.9^b^	8.1 ± 0.2^a^	14.6 ± 0.9^b^	10.2 ± 0.2^a^	14.6 ± 0.5^b^	9.4 ± 0.3^a^	12.5 ± 0.7^b^	10.5 ± 0.2^a^	15.4 ± 1.5^b^
K^+^ (meq/L)	2.1 ± 0.2^a^	3.4 ± 0.9^b^	2.4 ± 0.2^a^	2.6 ± 0.4^b^	3.7 ± 0.2^a^	5.8 ± 0.5^b^	2.7 ± 0.6^a^	4.1 ± 0.8^b^	3.4 ± 0.7^a^	6.4 ± 1.5^b^
Na^+^ (meq/L)	10.1 ± 0.3^a^	15.6 ± 0.7^b^	11.3 ± 0.9^a^	15.6 ± 0.8^b^	14.1 ± 0.8^a^	17.1 ± 1.5^b^	10.8 ± 0.7^a^	16.1 ± 1.3^b^	13.3 ± 0.6^a^	14.8 ± 1.5^b^
Cl^+^ (meq/L)	21.3 ± 0.9^a^	20.4 ± 0.7^b^	17.5 ± 0.2^a^	16.5 ± 0.1^b^	14.6 ± 0.2^a^	18.4 ± 3.5^b^	22.1 ± 0.8^a^	24.5 ± 2.5^b^	20.7 ± 0.4^a^	24.3 ± 0.9^b^
Fe (ppm)	16.5 ± 1.0^a^	19.5 ± 2.0^b^	13.6 ± 0.2^a^	16.3 ± 0.8^b^	15.6 ± 0.8^a^	21.5 ± 2.5^b^	17.5 ± 1.6^a^	22.1 ± 1.5^b^	18.7 ± 0.2^a^	22.6 ± 0.9^b^
Mn (ppm)	15.6 ± 1.0^a^	17.5 ± 0.0^b^	16.3 ± 0.2^a^	19.5 ± 1.1^b^	21.7 ± 0.9^a^	26.4 ± 0.5^b^	16.3 ± 1.2^a^	19.7 ± 1.1^b^	15.4 ± 1.2^a^	20.5 ± 0.8^b^
Zn (ppm)	11.6 ± 1.1^a^	14.6 ± 1.1^b^	11.6 ± 0.2^a^	15.3 ± 0.9^b^	14.8 ± 1.2^a^	21.8 ± 0.5^b^	23.5 ± 1.3^a^	26.5 ± 1.3^b^	19.5 ± 2.0^a^	18.9 ± 1.5^b^
Cu (ppm)	9.5 ± 0.7^a^	12.6 ± 1.0^b^	10.3 ± 0.2^a^	11.5 ± 1.9^b^	9.4 ± 1.6^a^	10.2 ± 0.9^b^	12.3 ± 0.7^a^	11.3 ± 0.8^b^	10.6 ± 0.2^a^	9.8 ± 0.1^b^
P (ppm)	35.4 ± 2.3^aa^	40.2 ± 0.9^b^	46.6 ± 0.2^a^	52.3 ± 1.5^b^	36.1 ± 1.8^a^	44.5 ± 0.9^b^	45.6 ± 2.6^a^	49.5 ± 0.7^b^	51.1 ± 2.2^a^	65.3 ± 3.5^b^
N (ppm)	100.3 ± 5.7^a^	120 ± 6.1^b^	113 ± 5.2^a^	129.4 ± 7.5^b^	111.7 ± 7.2^a^	192 ± 6.5^b^	132.4 ± 5.2^a^	170.9 ± 4.5^b^	115.7 ± 9.8^a^	148.5 ± 6.9^b^
T. Phen	90.1 ± 4.7^a^	110 ± 6.8^b^	83 ± 5.9^a^	113.2 ± 7.0^b^	91.2 ± 4.4^a^	172.8 ± 6.3^b^	132.1 ± 5.2^a^	158.0 ± 4.2^b^	101.9 ± 7.2^a^	138.4 ± 6.3^b^
OM (%)	1.3 ± 0.1^a^	1.95 ± 0.0^b^	1.15 ± 0.2^a^	1.92 ± 0.5^b^	1.4 ± 0.2^a^	2.18 ± 0.6^b^	1.09 ± 0.2^a^	1.66 ± 0.3^b^	1.28 ± 0.2^a^	2.04 ± 0.2^b^
Yield of the trees					
Kg/tree	88.5 ± 3.2^a^	99.6 ± 5.4^b^	75.3 ± 7.2^a^	89.4 ± 6.2^b^	83.2 ± 5.5^a^	92.4 ± 0.5^b^	113.5 ± 9^a^	133.7 ± 3.6^b^	103.2 ± 5.9^a^	122.1 ± 4.3^b^

*Values are mean ± standard error of three independent replicates. Significant differences (Student’s t-test *P* < 0.05) are marked with different letters (a, b) within the same cultivar. Data are averages ± SE (n = 5).*

Incorporation of vermicompost in the soil had a significant positive impact on the yield of date palm fruits, with increases of 9 to 15%, and the highest increases were in the Ajwa and Ruthana cultivars (Table 1). Such an enhancement in the yield may be a logical consequence of the improved soil fertility due to the release of nutrients during vermicompost decomposition. Additionally, vermicompost incorporation in the soil is believed to improve water retention, nutrient uptake and photosynthesis (Tan and Hogan, 1995; Tanaka et al., 1998; Theunissen et al., 2010). The higher productivity of plants grown in fertilized soils may also be attributed to the increased availability of phenolic compounds and mineral contents of the decomposed residue, which can reduce the susceptibility of plants to phytopathogens and increase photosynthetic reactions (Theunissen et al., 2010). In this context, high availability of microorganisms such as N-fixing bacteria in the fertilized soil with vermicompost can produce growth-promoting compounds such as plant hormone analogs and growth regulators leading to increased plant growth and yield (Theunissen et al., 2010).

### Vermicompost Differentially Accumulated Primary Metabolites Among the Different Cultivars of Date Palm

#### Sugars Profile

**FIGURE 1:**
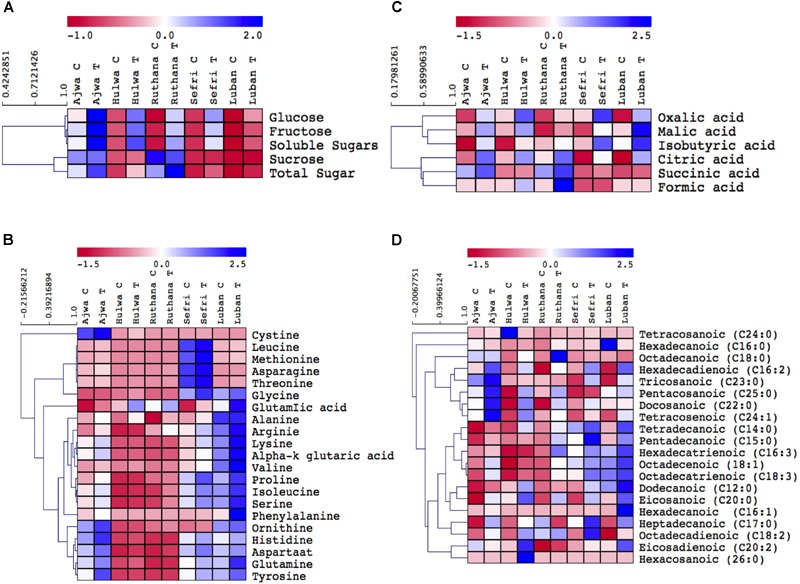
Hierarchical clustering of primary metabolites [sugars **(A)**, amino acids **(B)**, organic acids **(C)**, and fatty acids **(D)**] concentrations in vermicompost-fertilized and non-fertilized *P. dactylifera* cv. Ajwa, Hulwa, Ruthana, Sefri, and Luban fruits. Vermicompost fertilization was carried out at a rate of 10 Kg/tree and fruits were harvested at the tamar stage.

Date flesh is a high-energy food due to its high sugar contents, particularly glucose and fructose (Khalid et al., 2017). For instance, 33 and 74% of Ajwa date are sugars. In our analysis, the highest and lowest levels of glucose, fructose and total soluble sugars were observed in Ajwa and Luban fruits under control conditions, respectively (Figure 1A and Supplementary Table S1). Sucrose and total insoluble sugars, on the other hand, showed a somewhat different profile with a strong increase in Ruthana. Notably, in most sugars, vermicompost fertilization caused a strong increase in sugar concentration in all date varieties and particularly in Ajwa, Hulwa, and Ruthana. Luban fruits showed the lowest sugars levels under control and vermicompost fertilization conditions. Sucrose level, in contrast, decreased in Ruthana date palm fruits after vermicompost treatment. Uptake of the plant macro-nutrients such as N, K, P, and Mg provided by vermicompost supply, is involved in the chlorophyll biosynthesis and hence increased photosynthesis (Tanaka et al., 1998). The high rate of photosynthesis increased carbon input into fruits, which could result in the induced sugars levels (Theunissen et al., 2010).

#### Amino Acids Profile

The amino acids analysis of date palm fruits showed a higher percentage of essential amino acids (Hamad et al., 2015). Amino acids are precursors for protein synthesis and a few amino acids, such as proline, act as antioxidants. We quantified 21 amino acids (Figure 1B and Supplementary Table S2), and, when looking at the concentration profiles at a glance, it is immediately clear that some profiles have very similar characteristics. For example, the profiles of glutamine, histidine, aspartate, and tyrosine are accumulated in Ajwa, Sefri and Luban and they further increased by vermicompost fertilization. Other amino acids (leucine, methionine, asparagine, and threonine), showed the highest levels irrespective to vermicompost fertilization in Sefri date fruits. Also, Sefri and Luban contained higher levels of the rest of the amino acids, particularly after fertilization, except for cysteine, which was accumulated in Ajwa date fruits.

These results suggested that, like sugars, vermicompost treatments induced accumulation of amino acids and these effects were date cultivar-dependent. Similarly, the study of Manivannan et al. (2009) indicated that amino acid contents were increased in crops grown under vermicompost application when compared to control. Vermicompost directly enriched the soil with nitrogen or indirectly by enhancing N-fixing bacteria (Theunissen et al., 2010). Moreover, vermicompost incorporated chemical antagonists such as amino acids into the soil (Nagavallemma et al., 2004). Consequently, under vermicompost fertilization, nitrogen uptake by date palm trees was markedly incorporated in amino acids biosynthesis and could explain the higher levels of amino acids in the date fruits (Theunissen et al., 2010).

#### Organic Acids Profile

The presence of organic acids not only increases the fruits’ nutritive value but also improves their sensory characteristics (e.g., sour, tart, and acidic). For instance, malic acid is one of the abundant organic acids in date fruits and significantly contributes to the characteristic fruity, smooth and sour taste of the fruits (Khalid et al., 2017). Organic acids also prolonged the quality of fruits because they reduced the growth of microorganisms in the fruit. Here, we also analyzed organic acid contents in the target date fruits, focusing on the changes in their concentrations after vermicompost fertilization (Figure 1C and Supplementary Table S3). Heat map representations showed an increase in the organic acids oxalic, malic, and isobutyric in Ajwa, Hulwa, Sefri, and Luban cultivars, and to lesser extent in Ruthana. On the other hand, Ruthana significantly accumulated citric, succinic and formic acids and higher inductions were also recorded for other cultivars of date palm after vermicompost application. Increased carbon assimilation in vermicompost-fertilized plants resulted in high carbon input in fruits, and that could explain the increase in metabolite levels such as organic and FA (Theunissen et al., 2010). Moreover, organic acid content and composition may be affected by experimental growth conditions, soil fertilization and fruit maturity (Ahmed et al., 1995).

#### Fatty Acids Profile

In date palm fruits, lipid content represents about 10% of dry mass (Galeb et al., 2012). For instance, Ajwa fruits have good lipid quality indices where about 21 and 75% of the total fatty acids (FA) are saturated fatty acids (SFA) and unsaturated fatty acids (UFA), respectively (Galeb et al., 2012). To reveal the impact of vermicompost amendment on the FA composition of date palm fruits, SFA and UFA were analyzed by GC/MS analysis (Figure 1D and Supplementary Table S4). Date palm varieties differentially accumulated FA under control conditions, for instance, Ajwa, Hulwa and Luban showed increased levels of tricosanoic, tetracosanoic, and hexadecanoic acids, respectively. The first profile observed for FA contains those which increased in all varieties and particularly in Ajwa date fruits by vermicompost application, and is almost uniquely composed of unsaturated and saturated FA (C16:2, C23:0, C25:0, C22:0, C24:1). The second profile showed these FA highly increased in fertilized Sefri, Hulwa and/or treated Luban (C14:0, C15:0, C16:3, C18:1, C18:3, C12:0, C20:0, C16:1, C17:0, C18:2, C20:2, C26:0). In contrast, another profile consisted of the FA which decreased during vermicompost treatment and contains mostly individual saturated FA (C24:0, C18:0 and C16:0, for Hulwa, Ruthana and Luban; respectively). Our results are similar to the results of Al-Shahib and Marshall (2003), who reported differences in fatty acid contents between 14 varieties of date palm fruits which were ascribed to varietal differences. The results of this study suggested that treated date palm fruits are rich sources of edible oils for human consumption.

### Vermicompost Supply Increased the Nutritive Values of the Fertilized Date Palm Fruits

#### Antioxidant Phenolic and Flavonoid Compounds

**FIGURE 2:**
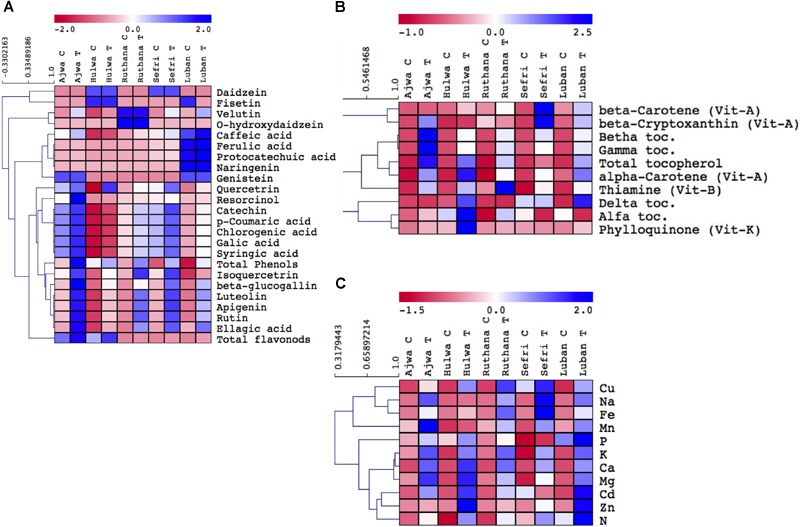
Hierarchical clustering of phenolics and flavonoids **(A)**, vitamin **(B)**, and mineral **(C)** concentrations in vermicompost-fertilized and non-fertilized *P. dactylifera* cv. Ajwa, Hulwa, Ruthana, Sefri, and Luban fruits. Vermicompost fertilization was carried out at a rate of 10 Kg/tree and fruits were harvested at the tamar stage.

A high content of total phenolic and flavonoids was recorded in non-treated date palm fruits in the range of 0.3–4 mg/g DW (Figure 2A and Supplementary Table S5). Because of the high content of polyphenolic compounds, date palm fruits are recommended as good antioxidant sources (Selim et al., 2012; Hamad et al., 2015). Our cluster analysis grouped the phenolics and flavonoids in different classes depending upon their responses. More specifically, treated Ajwa, and treated Sefri significantly accumulated quercetrin, resorcinol, catechol, p-coumaric acid, chlorogenic acid, galic acid, syringic acid, isoquercitrin, beta glucogallin, luteolin, apigenin, rutin, and ellagic acid. On the other hand, treated Ruthana contained high levels of velutin and O-hydroxydaizein, while Luban contained high levels of caffeic acid, ferulic acid, protocatechuic acid, and naringenin. Due to the slow and gradual release of plant-available nutrients from vermicompost, higher levels of total phenolic compounds were observed in vermicompost-treated plants (Theunissen et al., 2010). More specifically, the high humic acid levels in vermicompost could be involved in the synthesis of phenolic compounds, which may improve plant quality (Theunissen et al., 2010). High phenolic content also acts as a deterrent to pests and diseases. The heterogeneity of these results could be attributed to several factors including date palm cultivar and growing conditions (Hamad et al., 2015). Finally, it is highly important to understand how vermicomposting fertilization should be manipulated to induce the highest phenolic level, which grants an improvement in antioxidant and nutritive quality.

#### Vitamins

Date palm fruits are good sources for vitamins such as Vit-C (L-ascorbic acid), Vit-A (carotene), Vit-B1 (thiamine), and Vit-B2 (riboflavin) (Al-Shahib and Marshall, 2003). Beside these vitamins, we also detected considerable levels of tocopherols, phylloquinone (Vit-K) and beta-cryptoxanthine (Vit-A) (Figure 2B and Supplementary Table S6). Untreated Ruthana and Luban cultivars showed increased levels of Vit-B1 and alpha-tocopherol, respectively. High contents of pro-vitamin A and vitamin C were recorded for some date palm cultivars such as Ajwa (Khalid et al., 2017). After vermicomposting, Ajwa showed the highest levels of beta and gamma tocopherols, however, Hulwa and Luban cultivars contained the highest levels of total tocopherol, Vit-K and beta-carotene, respectively. Similarly, vermicompost fertilization was reported to increase concentrations of vitamins such as ascorbate (Vit-C) (Theunissen et al., 2010). In this regard, previous studies indicated that nutrients, such as nitrogen, released from vermicompost could explain the increased concentration of vitamins such as carotene (Mozafar, 1993). Also, it induced vitamin levels that could be attributed to increased carbon assimilation (high photosynthetic rate). Therefore, applying vermicompost will not only enhance the plant yield but also its nutritive quality.

#### Mineral Profiling

In general, significant amounts of minerals in date palm fruits were observed, and they were significantly induced by vermicompost treatment (Figure 2C and Supplementary Table S7). In particular, potassium and phosphorus contents were the highest (0.01–0.2 mg/g DW). Previously, it was reported that mineral contents in date palm fruits were more than three to five times those compared to their amounts in grapes, apples, oranges and bananas (Al-Showiman, 1998). In our study, induced levels of Mn in Ajwa; Ca and Mg in Hulwa; Cu, Na, and Fe in Sefri; P, Cd, and Zn in Luban; and N in the fruits of all tested cultivars were reported. Similarly, foliar application of vermicompost was reported to increase mineral contents (Tejada et al., 2007). Therefore, date palm fruits of Hulwa and Sefri cultivars grown in soil amended with vermicompost could be used as good sources of macronutrients essential for the development of bone, production of red blood cells and energy metabolism (Fahad et al., 2014). Induced levels of nutrients could be explained by the fact that vermicompost released both macro and micronutrients in the treated soil. Absorption of minerals on humic acid makes them available for plant growth (Gutiérrez-Miceli et al., 2007). High nutrient level was not only due to nutrient uptake, but also to the plant’s ability to assimilate essential nutrients under vermicompost application (Theunissen et al., 2010). The high level of K and the low level of Na in most of the tested date palm fruits are useful for people with hypertension (Khalid et al., 2017). Thus, checking the nutrient constitution of vermicompost samples before their use will provide useful information on the released nutrient types and quality.

### Vermicompost Fertilization Induced the Medicinal Activities of Date Palm Fruits Ethanol Extracts

#### Antioxidant, Anticancer, Antibacterial, and Antiprotozoal Activities

**FIGURE 3:**
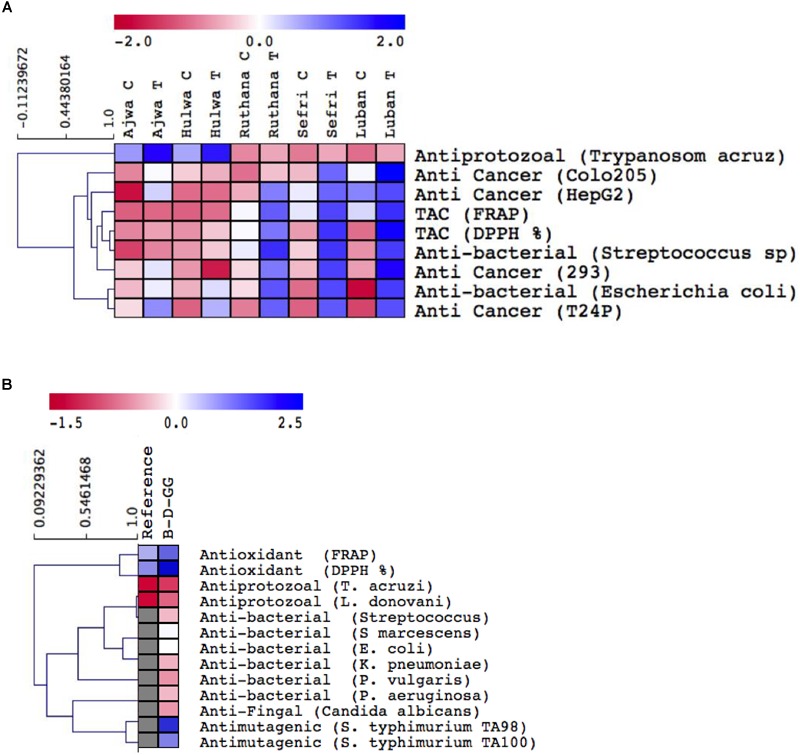
Hierarchical clustering medicinal bioactivities of the ethanol extracts **(A)** of vermicompost-fertilized and non-fertilized *P. dactylifera* cv. Ajwa, Hulwa, Ruthana, Sefri, and Luban fruits and β-D-glucogallin **(B)**. Vermicompost fertilization was carried out at a rate of 10 Kg/tree and fruits were harvested at the tamar stage.

Date palm fruits are rich in phytochemicals and have been used to treat human diseases and were reported to possess anti-inflammatory, antioxidant and anticancer activities (Hussain et al., 2014; Khalid et al., 2017). Therefore, we evaluated different biological activities (antioxidant, antibacterial, antiprotozoal, and anticancer) of the ethanol extracts of the selected date palm fruits (Figure 3A and Supplementary Table S8). Overall, the ethanol extracts from all the treated varieties of date palm fruits showed increased antioxidant levels, antibacterial, antiprotozoal, and/or anticancer abilities (Figure 3A). In particular, Ajwa and Hulwa cultivars showed the strongest antiprotozoal capacity under vermicompost fertilization, whereas Ruthana, Sefri and Luban exhibited high anticancer (Colo205, HepG2, T24P, and 293), antioxidant (FRAP and DPPH%) and/or antibacterial activities against *Streptococcus spp*. and *E. coli*. Antioxidant and anticancer capacities of the ethanol extracts of date palm fruits could be attributed to the availability of antioxidants such as phenolics, flavonoids, steroids, tocopherols, and ascorbate (Hussain et al., 2014). Induced antioxidants prevent free radical accumulation, which could damage cellular molecules, resulting in cancer and aging-related diseases (Al-Alawi et al., 2017). On the other hand, date palm fruit extract also showed induced antioxidative enzyme activities such as catalase enzyme, which is involved in proliferative damage inhibition (Al-Alawi et al., 2017). In the study of Al-Yahya et al. (2015), date extracts delayed the depletion of vital antioxidant enzymes like carnitine acyl transferase and superoxide dismutase.

Moreover, the increased phenolics and flavonoid contents in date palm fruits resulted in improved antimicrobial activity (Hussain et al., 2014). For example, date extracts inhibited the activity of many Gram-positive and Gram-negative bacteria such as *Bacillus cereus, S. marcescens, S. aureus, E. coli*, and *K. pneumonia* (Aamir et al., 2013). The extract of *P. dactylifera* cv. Ajwa fruits also showed significant positive effects on the causative pathogen of enteric diseases, since it inhibited *Enterococcus faecalis* growth (Aamir et al., 2013). We therefore proposed the treated date palm fruits as a good and inexpensive source for natural products to control microbial infectious diseases.

### Principal Component Analysis (PCA) Supported the Cultivars Specificity

**FIGURE 4:**
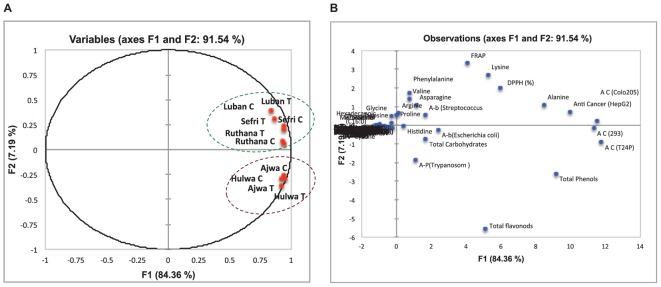
Principal component analysis (PCA) of the measured metabolites, mineral concentrations and bioactivities of the ethanol extracts of vermicompost-fertilized and unfertilized date fruits of *P. dactylifera* cv. Ajwa, Hulwa, Ruthana, Sefri, and Luban **(A)**. PCA-plot separating the date palm cultivars and **(B)** the measured metabolites, mineral concentrations, and bioactivities.

To summarize the differences between date palm cultivar responses at the metabolic and biological activity levels, and to independently test the deductions of cluster analysis, all data were subjected to PCA (Figure 4A). PCA clearly separated the cultivars into two different groups, the first two components (PC1 and PC2) together explaining 91% of the variability. The first group consisted of the cultivars Luban, Sefri, and Ruthana and the second group consisted of the cultivars Ajwa and Hulwa. PC1 (83.36) was heavily loaded on parameters related to medicinal activities (e.g., antioxidants, anticancer, antiprotozoal, and antibacterial activity), antioxidants (e.g., phenols and flavonoids) and amino acids (proline, valine, asparagine, and alanine) (Figure 4). On the other hand, no separation according to fertilizer treatments was recorded. PCA data also indicated that bioactivities of the fruit extracts and their antioxidants tend to display correlated output values. For instance, anticancer activity, antibacterial activity, antioxidants, phenolic, and flavonoids were grouped together.

### Medical Bioactivity of the Isolated β-D-Glucogallin

It is well known that phenolic compounds possess antioxidant, antimicrobial and anti-inflammatory activities (Hamad et al., 2015). Among the phenolic compounds which were increased by vermicompost application is β-D-glucogallin. β-D-glucogallin is widely distributed in angiosperms (Puppala et al., 2012). Under control and treatment conditions, many phenolic compounds were found in Ajwa fruits, and β-D-glucogallin represented the highest level. Therefore, β-D-glucogallin was isolated by chromatographic and spectroscopic methods for biological activity evaluation. β-D-glucogallin is well-known as an antioxidant and it showed anti-inflammatory and antitumor activities (Puppala et al., 2012). In addition, antibacterial and antifungal activities were also reported for β-D-glucogallin (Chanwitheesuk et al., 2007). β-D-glucogallin was extracted by TLC, where its spot on the TLC plate reacted with Folin–Ciocalteu’s reagent to produce a blue color. It was preliminarily identified by comparing its R_f_ value and retention time with those of authentic compounds. Finally, the structure of the isolated phenolic compound was elucidated based on IR, MS, and ^1^H and ^13^C NMR analyses.

Due to the emergence of drug-resistant bacteria, it is essential to investigate novel natural bioactive compounds with lesser resistance. Several studies attributed the antibacterial activity of plant extracts against bacterial pathogens to their phenolic composition (Ahmed et al., 2015). Therefore, investigating the structural diversity of phenolics in date palm fruit extracts could affect their exhibited antimicrobial potentials. Here, the bioactivities of phenolic acids extracted from date palm extracts against different bacterial strains were studied. Standard disk diffusion technique analyses indicated the strong antibacterial potential of β-D-glucogallin (Figure 3B, Supplementary Figure S1, and Supplementary Table S9). Varying degree of microorganism sensitivity to β-D-glucogallin treatment was also documented, to suggest that there was differential intrinsic tolerance of microorganisms to β-D-glucogallin. Strong and weak antimicrobial activities of β-D-glucogallin were recorded against *S. aureus*, and *Candida albicans*, respectively. Meanwhile, no inhibitory effect was noticed against *S. marcescens, P. vulgaris*, and *P. aeruginosa*. The bacterial physiological indexes MIC and MBC were studied (Supplementary Table S8). The MIC and MBC values of date palm fruit extracts ranged from 125 to 500 μg/mL. β-D-glucogallin significantly inhibited the growth of *S. aureus* with a MIC value of 125 μg/ml. The Gram-positive bacteria were more susceptible to the phenolic acid than the Gram-negative bacteria. In a previous study of Tunon et al. (2009), it was found that tea flavonoids inhibited *Streptococcus mutans* and *Streptococcus sobrinus* at concentrations between 250 mg/mL and 1000 mg/ml.

Based on the promising antimicrobial activity, the anti-mutagenic activity was also investigated by the Ames test against the indirectly acting mutagen cyclophosphamide (Figure 3B and Supplementary Table S10). It has been hypothesized that bio-antimutagens act as second stage inhibitors that block the mutagen before they could attack the DNA (Hung et al., 2009). The anti-mutagenic activity of β-D-glucogallin was evaluated against the TA98 and TA100 strains of *S. typhimurium*. Significant anti-mutagenic activity (*p* < 0.01) of β-D-glucogallin under all applied concentrations was recorded. More specifically, β-D-glucogallin showed higher anti-mutagenic activity at a concentration of 2500 μg/plate against *S. typhimurium* TA98. For both tested bacterial strains, mutagenicity decreased from 45 to 5 for TA98 and from 36 to 12 for TA100. The induced anti-mutagenic activity of β-D-glucogallin could be attributed to its powerful radical scavenger activity. β-D-glucogallin reduces the mutagenicity caused by indirect acting mutagen cyclophosphamide by 45 and 36%, respectively, in the strains TA98 and TA100 at the highest tested dose (2500 g/plate), which proves the strong anti-mutagenic activity. It also had strong and effective anti-mutagenicity against cyclophosphamide. With respect to the antiprotozoal activity, β-D-glucogallin showed high activity against *T. cruzi* and *L. donovani* (Figure 3B and Supplementary Table S11). IC_50_ values of β-D-glucogallin were 4.8 and 8.6 μg/mL, respectively. In general, high polarity of β-D-glucogallin could explain its antiprotozoal activity. Similar to antiprotozoal activity, the DPPH scavenging activity and total antioxidant capacity (FRAP) were also recorded for β-D-glucogallin (Figure 3B and Supplementary Table S12). DPPH radical-scavenging activity and FRAP of β-D-glucogallin extracted from date palm fruits were higher than the one recorded for gallic acid (49.2 and 39.4 for DPPH and FRAP, respectively).

## Conclusion

The findings of the present work suggested that fertilizing palm date fruits with vermicompost is a promising eco-friendly approach to improve date palm fruit yield, nutritive quality and biological activities. Vermicompost improved soil health and fertility by increasing nutrients, plant growth regulators and humic acids. Improved soil fertility could explain the high health-beneficial value of the produced date palm fruits as indicated by the elevated levels of primary metabolites, minerals, vitamins, essential amino acids, unsaturated FA and antioxidants. Consequently, increased amounts of compounds that are beneficial to health enhanced the medicinal value of date fruit ethanol extracts. Our results also signify that *P. dactylifera* cv. Ajwa date fruit extract was a good source of the natural bioactive compound β-D-glucogallin, which showed significant antioxidant activity as well as promising action against microbial and protozoal growth.

## Author Contributions

SAJ, SS, SH, WH, HA, and HAE set-up the experiments. HAE, SS, and HM did the analyses and HAE, HA, and WH wrote the manuscript. All authors revised and approved the manuscript.

## Conflict of Interest Statement

The authors declare that the research was conducted in the absence of any commercial or financial relationships that could be construed as a potential conflict of interest.

## Footnotes

1 http://gmd.mpimp-golm.mpg.de

## Supplementary Material

FIGURE S1 Effect of different concentrations of β-D-glucogallin, 0 (control, ◆), 100 μg/ml (■), 200 μg/ml (▲) on the viability of *Staphylococcus aureus*. Values are the average of three individual replicates (means ± SD). Different letters represent significant differences between the treatments (Duncan test; *P* < 0.05 and *n* = 4).

TABLE S1 Sugar concentrations in vermicompost-fertilized and non-fertilized *P. dactylifera* cv. Ajwa, Hulwa, Ruthana, Sefri, and Luban fruits. Vermicompost fertilization was carried out at a rate of 10 Kg/tree and fruits were harvested at the tamar stage. Different letters represent significant differences between the treatments in each cultivar (Duncan test; *P* < 0.05 and *n* = 4).

TABLE S2 Amino acid concentrations in vermicompost-fertilized and non-fertilized *P. dactylifera* cv. Ajwa, Hulwa, Ruthana, Sefri, and Luban fruits. Vermicompost fertilization was carried out at a rate of 10 Kg/tree and fruits were harvested at the tamar stage. Different letters represent significant differences between the treatments in the cultivar (Duncan test; *P* < 0.05 and *n* = 4).

TABLE S3 Organic acid concentrations in vermicompost-fertilized and non-fertilized *P. dactylifera* cv. Ajwa, Hulwa, Ruthana, Sefri, and Luban fruits. Vermicompost fertilization was carried out at a rate of 10 Kg/tree and fruits were harvested at the tamar stage. (a) Heat map and cluster tree representation of the organic acids, (b) the measured metabolites. Different letters represent significant differences between the treatments in the cultivar (Duncan test; *P* < 0.05 and *n* = 4).

TABLE S4 Fatty acid concentrations in vermicompost-fertilized and non-fertilized *P. dactylifera* cv. Ajwa, Hulwa, Ruthana, Sefri, and Luban fruits. Vermicompost fertilization was carried out at a rate of 10 Kg/tree and fruits were harvested at the tamar stage. Different letters represent significant differences between the treatments in the cultivar (Duncan test; *P* < 0.05 and *n* = 4).

TABLE S5 Mineral concentrations in vermicompost-fertilized and non-fertilized *P. dactylifera* cv. Ajwa, Hulwa, Ruthana, Sefri, and Luban fruits. Vermicompost fertilization was carried out at a rate of 10 Kg/tree and fruits were harvested at the tamar stage. Different letters represent significant differences between the treatments in the cultivar (Duncan test; *P* < 0.05 and *n* = 4).

TABLE S6 Vitamin concentrations in vermicompost-fertilized and non-fertilized *P. dactylifera* cv. Ajwa, Hulwa, Ruthana, Sefri, and Luban fruits. Vermicompost fertilization was carried out at a rate of 10 Kg/tree and fruits were harvested at the tamar stage. Different letters represent significant differences between the treatments in the cultivar (Duncan test; *P* < 0.05 and *n* = 4).

TABLE S7 Phenolics and flavonoid compound concentrations in vermicompost-fertilized and non-fertilized *P. dactylifera* cv. Ajwa, Hulwa, Ruthana, Sefri, and Luban fruits. Vermicompost fertilization was carried out at a rate of 10 Kg/tree and fruits were harvested at the tamar stage. Different letters represent significant differences between the treatments in the cultivar (Duncan test; *P* < 0.05 and *n* = 4).

TABLE S8 Medicinal bioactivities of the ethanol extracts of vermicompost-fertilized and non-fertilized *P. dactylifera* cv. Ajwa, Hulwa, Ruthana, Sefri, and Luban fruits. Vermicompost fertilization was carried out at a rate of 10 Kg/tree and fruits were harvested at the tamar stage. Different letters represent significant differences between the treatments in the cultivar (Duncan test; *P* < 0.05 and *n* = 4).

TABLE S9 Antimicrobial activity of β-D-glucogallin (100 μg/disc) extracted from Ajwa date fruits. Values are expressed as average of three replicates ± SD. Different letters represent significant differences between the treatments in the same raw (Duncan test; *P* < 0.05 and *n* = 4).

TABLE S10 Antimutagenic potential of β-D-glucogallin against *S. typhimurium* TA98 and TA100 bacterial strains. Values are expressed as the average of three replicates ± SD. Different letters represent significant differences between the treatments in the same raw (Duncan test; *P* < 0.05 and *n* = 4).

TABLE S11 Antiprotozoal activity of the investigated β-D-glucogallin against *Trypanosoma cruzi* and *Leishmania donovani*. Activities are presented as IC_50_ μg/mL (Standard compounds: Benznidazole^a^ and Miltefosine^b^). Values are expressed as the average of three replicates ± SD. Different letters represent significant differences between the treatments in the same raw (Duncan test; *P* < 0.05 and *n* = 4).

TABLE S12 Antioxidant capacity using DPPH and FRAP assays of the isolated β-D-glucogallin. DPPH: 2,2-diphenyl-1-picrylhydrazyl, FRAP: ferric reducing antioxidant power. Values are expressed as the mean of three replicates ± SD. Different letters represent significant differences between the treatments in the same raw (Duncan test; *P* < 0.05 and *n* = 4).

## References

[B1] Aamir J. Kumari A. Khan M. N. Medam S. K. (2013). Evaluation of then combinational antimicrobial effect of *Annona Squamosa* and *Phoenix Dactylifera* seeds methanolic extract on standard microbial strains. *Int. J. Biol. Sci.* 2 68–73.

[B2] AbdElgawad H. Farfan-Vignolo E. R. de Vos D. Asard H. (2015). Elevated CO2 mitigates drought and temperature-induced oxidative stress differently in grasses and legumes. *Plant Sci.* 231 1–10. 10.1016/j.plantsci.2014.11.001 25575986

[B3] AbdElgawad H. Peshev D. Zinta G. Van den Ende W. Janssens I. A. Asard H. (2014). Climate extreme effects on the chemical composition of temperate grassland species under ambient and elevated CO2: a comparison of fructan and non-fructan accumulators. *PLoS One* 9:e92044. 10.1371/journal.pone.0092044 24670435 PMC3966776

[B4] Ahmed D. Khan M. M. Saeed R. (2015). Comparative analysis of phenolics, flavonoids, and antioxidant and antibacterial potential of methanolic, hexanic and aqueous extracts from *Adiantum caudatum* leaves. *Antioxidants* 4 394–409. 10.3390/antiox4020394 26783712 PMC4665467

[B5] Ahmed I. A. Ahmed A. W. K. Robinson R. K. (1995). Chemical composition of date varieties as influenced by the stage of ripening. *Food Chem.* 54 305–309. 10.1016/0308-8146(95)00051-J 19492811

[B6] Al-Alawi R. A. Al-Mashiqri J. H. Al-Nadabi J. S. M. Al-Shihi B. I. Baqi Y. (2017). Date palm tree (*Phoenix dactylifera* L.): natural products and therapeutic options. *Front. Plant Sci.* 8:845. 10.3389/fpls.2017.00845 28588600 PMC5440559

[B7] Al-Farsi M. Lee C. Y. (2008). Optimization of phenolics and dietary fibre extraction from date seeds. *Food Chem.* 108 977–985. 10.1016/j.foodchem.2007.12.009 26065761

[B8] Al-Shahib W. Marshall R. J. (2003). The fruit of the date palm: it’s possible use as the best food for the future. *Int. J. Food Sci. Nutr.* 54 247–259. 10.1080/09637480120091982 12850886

[B9] Al-Showiman S. S. (1998). *Al Tamr, Ghetha wa Saha (Date, Food and Health).* Qassim: Dar Al Khareji Press.

[B10] Alu’datt M. H. Rababah T. Alhamad M. N. Al-Mahasneh M. A. Almajwal A. Gammoh S. (2017). A review of phenolic compounds in oil-bearing plants: distribution, identification and occurrence of phenolic compounds. *Food Chem.* 218 99–106. 10.1016/j.foodchem.2016.09.057 27719963

[B11] Al-Yahya M. Raish M. AlSaid M. S. Ahmad A. Mothana R. A. Al-Sohaibani M. (2015). Ajwa’dates (*Phoenix dactylifera L.*) extract ameliorates isoproterenol-induced cardiomyopathy through downregulation of oxidative, inflammatory and apoptotic molecules in rodent model. *Phytomedicine* 23 1240–1248. 10.1016/j.phymed.2015.10.019 26776662

[B12] Ames B. N. McCann J. Yamasaki E. (1975). Methods for detecting carcinogens and mutagens with the Salmonella/mammalian microsome mutagenicity test. *Mutat. Res.* 31 347–364. 10.1016/0165-1161(75)90046-1 768755

[B13] Assirey E. A. (2015). Nutritional composition of fruit of 10 date palm (*Phoenix dactylifera* L.) cultivars grown in Saudi Arabia. *J. Taibah Univ. Sci.* 9 75–79. 10.1016/j.jtusci.2014.07.002

[B14] Chanwitheesuk A. Teerawutgulrag A. Kilburn J. D. Rakariyatham N. (2007). Antimicrobial β-D-glucogalline from *Caesalpinia* mimosoides Lamk. *Food Chem.* 100 1044–1048. 10.1016/j.foodchem.2005.11.008

[B15] Domínguez J. (2004). “State of the art and new perspectives on vermicomposting research,” in *Earthworm Ecology* 2nd Edn ed. Edwards C. A. (Boca Raton, FL: CRC Press) 401–424.

[B16] Fahad A. L. Juhaimi K. G. Özcan M. M. (2014). Physicochemical properties and mineral contents of seven different date fruit (*Phoenix dactylifera* L.) varieties growing in Saudi Arabia. *Environ. Monit. Assess.* 186 2165–2170. 10.1007/s10661-013-3526-3 24346347

[B17] Galeb H. A. Salimon J. Eid E. E. M. Nacer N. E. Saari N. Saadi S. (2012). The impact of single and double hydrogen bonds on crystallization and melting regimes of ajwa and barni lipids. *Food Res. Int.* 48 657–666. 10.1016/j.foodres.2012.06.006

[B18] Gupta R. Yadav A. Garg V. K. (2014). Influence of vermicompost application in potting media on growth and flowering of marigold crop. *Int. J. Recycl. Org. Waste Agric.* 3 41–47. 10.1007/s40093-014-0047-1

[B19] Gutiérrez-Miceli F. A. Santiago-Boraz J. Molina J. A. M. Nafate C. C. Abdul-Archila M. Llaven M. A. O. (2007). Vermicompost as a soil supplement to improve growth, yield and fruit quality of tomato (*Lycopersicum esculentum*). *Bioresour Technol.* 98 2781–2786. 10.1016/j.biortech.2006.02.032 17400447

[B20] Hamad I. AbdElgawad H. Al Jaouni S. Zinta G. Asard H. Hassan S. (2015). Metabolic analysis of various date palm fruit (*Phoenix dactylifera* L.) cultivars from Saudi Arabia to assess their nutritional quality. *Molecules* 20 13620–13641. 10.3390/molecules200813620 26225946 PMC6331958

[B21] Hung Y. H. Wang Y. J. Chou C. C. (2009). Antimutagenic activity of *Aspergillusawamori*-fermented black soyabean response to stimulated digestive juice treatments and its antimutagenic mechanisms. *LWT-Food Sci. Technol.* 42 56–62. 10.1016/j.lwt.2008.06.001

[B22] Hussain T. M. Qadir M. I. Ali M. Ahmad B. Khan Y. H. (2014). Ajwa date (*Phoenix dactylifera*): an emerging plant in pharmacological research. *Pak. J. Pharm. Sci.* 27 607–616. 24811825

[B23] Jakob E. Elmadfa I. (1996). Application of a simplified HPLC assay for the determination of phylloquinone (vitamin K1) in animal and plant food items. *Food Chem.* 56 87–91. 10.1016/0308-8146(95)00150-6

[B24] Jones J. (2001). *Laboratory Guide for Conducting Soil Tests and Plant Analysis.* Boca Raton, FL: CRC press.

[B25] Khalid S. Khalid N. Khan R. S. Ahmed H. Ahmad A. (2017). A review on chemistry and pharmacology of Ajwa date fruit and pit. *Trends Food Sci. Technol.* 63 60–69. 10.1016/j.tifs.2017.02.009

[B26] Manivannan S. Balamurugan M. Parthasarathi K. Gunasekaran G. Ranganathan L. S. (2009). Effect of vermicompost on soil fertility and crop productivity of beans (*Phaseolus vulgaris*). *J. Environ. Biol.* 30 275–281. 20121031

[B27] Mozafar A. (1993). Nitrogen fertilizers and the amount of vitamins in plants: a review. *J. Plant Nutr.* 16 2479–2506. 10.1080/01904169309364698

[B28] Nagavallemma K. P. Wani S. P. Lacroix S. Padmaja V. V. Vineela C. BabuRao M. (2004). *Vermicomposting: Recycling Wastes into Valuable Organic Fertilizer. Global Theme on Agrecosystems* Report no. 8. Patancheru 502 324. Andhra Pradesh: International Crops Research Institute for the Semi-Arid Tropics.

[B29] O’Connell P. F. (1992). Sustainable agriculture-a valid alternative. *Outlook Agric.* 21 5–12. 10.1177/003072709202100103

[B30] Puppala M. Ponder J. Suryanarayana P. Reddy G. B. Petrash M. LaBarbera D. V. (2012). The isolation and characterization of β-glucogallin as a novel aldose reductase inhibitor from *Emblica officinalis*. *PLoS One* 7:e31399. 10.1371/journal.pone.0031399 22485126 PMC3317655

[B31] Rajan V. K. Muraleedharan K. (2017). A computational investigation on the structure, global parameters and antioxidant capacity of a polyphenol, Gallic acid. *Food Chem.* 220 93–99. 10.1016/j.foodchem.2016.09.178 27855941

[B32] Räz B. Iten M. Grether-Bühler Y. Kaminsky R. Brun R. (1997). The Alamar blue assay to determine drugs sensitivity of African trypanosomes (*Trypanosoma brucei rhodesiense* and *Trypanosoma brucei gambiense*). in vitro. *Acta Trop.* 68 139–147. 10.1016/S0001-706X(97)00079-X 9386789

[B33] Selim S. A. El Alfy S. Al-Ruwaili M. Abdo A. Al Jaouni S. (2012). Susceptibility of imipenem-resistant *Pseudomonas aeruginosa* to flavonoid glycosides of date palm (*Phoenix dactylifera* L.) tamar growing in Al Madinah. Saudi Arabia. *Afr. J. Biotech.* 11 416–422.

[B34] Siebert K. J. (1999). Effects of protein–polyphenol interactions on beverage haze, stabilization, and analysis. *J. Agric. Food Chem.* 47 353–362. 10.1021/jf980703o 10563900

[B35] Solowey E. Lichtenstein M. Sallon S. Paavilainen H. Solowey E. Lorberboum-Galski H. (2014). Evaluating medicinal plants for anticancer activity. *Sci. World J.* 2014:721402. 10.1155/2014/721402 25478599 PMC4248331

[B36] Subler S. Edwards C. A. Metzger J. D. (1998). Comparing vermicomposts and composts. *Biocycle* 39 63–66. 28390272

[B37] Tan W. Hogan D. G. (1995). Limitation to net photosynthesis as affected by nitrogen status in jackpine (*Pinusbanksiana Lamb.*) seedlings. *J. Exp. Bot.* 46 407–413. 10.1093/jxb/46.4.407

[B38] Tanaka A. Ito H. Tanaka R. Tanaka N. K. Yoshida K. Okada K. (1998). Chlorophyll a oxygenase (CAO) is involved in chlorophyll b formation from chlorophyll a. *Proc. Natl. Acad. Sci. U.S.A.* 95 12719–12723. 10.1073/pnas.95.21.12719 9770552 PMC22897

[B39] Tejada M. Gonzalez J. Hernandez M. Garcia C. (2007). Agricultural use of leachates obtained from two different vermicomposting processes. *Bioresour. Technol.* 99 6228–6232. 10.1016/j.biortech.2007.12.031 18215517

[B40] Theunissen J. Ndakidemi P. A. Laubscher C. P. (2010). Potential of vermicompost produced from plant waste on the growth and nutrient status in vegetable production. *Int. J. Phys. Sci.* 5 1964–1973.

[B41] Tucker P. (2005). *Co-Composting Paper Mill Sludges With Fruit and Vegetable Wastes.* Dissertation. University of Paisley Paisley.

[B42] Tunon M. J. Garcia-Mediavilla M. V. Sanchez-Campos S. Gonzales-Gallego J. (2009). Potential of flavonoids as anti-inflammatory: modulation of proinflammatory gene expression and signal transduction pathways. *Curr. Drug Metabol.* 10 256–271. 10.2174/138920009787846369 19442088

[B43] Van Oostende C. Widhalm J. R. Basset G. J. (2008). Detection and quantification of vitramin K1 quinol in leaf tissues. *Photochemistry* 69 2457–2462. 10.1016/j.phytochem.2008.07.006 18799171

