# Supplementary material for: Vermicompost Supply Modifies Chemical Composition and Improves Nutritive and Medicinal Properties of Date Palm Fruits From Saudi Arabia
Source: Front Plant Sci. 2019 Apr 11;10:424. doi: 10.3389/fpls.2019.00424 (PMC6470401; doi:10.3389/fpls.2019.00424)

|  |  |  |  |  | **Supplementary Table 1.**   \|  \| **Ajwa C** \| **Ajwa T** \| **Hulwa C** \| **Hulwa T** \| **Ruthana C** \| **Ruthana T** \| **Sefri C** \| **Sefri T** \| **Luban C** \| **Luban T** \| \| --- \| --- \| --- \| --- \| --- \| --- \| --- \| --- \| --- \| --- \| --- \| \| **Glucose** \| 0.77±0.01a \| 1.38±0.03b \| 0.62±0.00a \| 1.05±0.03b \| 0.53±0.01a \| 0.88±0.03b \| 0.59±0.01a \| 0.98±0.04b \| 0.44±0.0a \| 0.70±0.03b \| \| **Fructose** \| 1.01±0.04a \| 1.67±0.10a \| 0.73±0.01a \| 1.30±0.04b \| 0.66±0.00a \| 1.06±0.03a \| 0.67±0.02a \| 1.06±0.07a \| 0.49±0.02a \| 0.73±0.06b \| \| **Sucrose** \| 0.96±0.02a \| 1.06±0.011a \| 0.33±0.0a \| 0.38±0.05a \| 1.40±0.00a \| 1.18±0.03a \| 0.24±0.00a \| 0.25±0.01a \| 0.17±0.00a \| 0.16±0.02a \| \| **Soluble sugars** \| 2.15±0.01a \| 3.60±0.02ab \| 1.61±0.1a \| 3.03±0.15b \| 1.42±0.05a \| 2.50±0.12b \| 1.51±0.08a \| 2.24±0.21b \| 1.11±0.06a \| 1.64±0.16a \| \| **Total sugars** \| 30.50±0.7a \| 51.10±2.5b \| 13.3±0.1a \| 23.10±0.6b \| 38.80±0.06a \| 63.70±2.1b \| 10.8±0.01a \| 16.33±0.9b \| 7.01±0.15a \| 11.62±0.73b \|   **Supplementary Table 2.**   \|  \| **Ajwa C** \| **Ajwa T** \| **Hulwa C** \| **Hulwa T** \| **Ruthana C** \| **Ruthana T** \| **Sefri C** \| **Sefri T** \| **Luban C** \| **Luban T** \| \| --- \| --- \| --- \| --- \| --- \| --- \| --- \| --- \| --- \| --- \| --- \| \| **GlutamIic acid** \| 59.17±3.2a \| 77.46±4.2b \| 87.24±7.82a \| 120.29±10.8b \| 97.64±8.7a \| 115±10.3b \| 62.41±4.2a \| 87.8±5.9b \| 98.63±6.6a \| 152.14±1b \| \| **Glutamine** \| 365.36±20a \| 502.92±27.4b \| 89.82±5.9a \| 122.40±8.b \| 45.35±2.9a \| 59.92±3.9b \| 295.14±16.3a \| 418.5±23.1b \| 393.29±21.1a \| 678.61±37b \| \| **Lysine** \| 21.76±1.40a \| 31.59±2.10b \| 4.41±0.39a \| 5.034±0.45a \| 3.38±0.30a \| 4.81±0.43a \| 17.97±1.22a \| 31.28±9.12a \| 44.21±3.00a \| 72.44±4.92b \| \| **Alpha-k glutaric acid** \| 0.158±0.01a \| 0.23±0.01a \| 0.03±0.0a \| 0.04±0.00a \| 0.024±0.001a \| 0.031±0.0a \| 0.13±0.01a \| 0.17±0.01a \| 0.32±0.02a \| 0.52±0.03a \| \| **Histidine** \| 8.39±0.012a \| 12.16±0.81b \| 1.32±0.12a \| 1.70±0.15a \| 0.95±0.086a \| 1.01±0.1a \| 6.061±0.41a \| 8.04±0.54b \| 5.95±0.40a \| 7.85±0.53b \| \| **Alanine** \| 26.44±1.41a \| 32.36±2.01b \| 21.9±1.4a \| 31.31±1.01b \| 12.9±0.83a \| 26.93±1.7b \| 25.53±1.4a \| 30.64±1.7b \| 55.9±3.97a \| 58.27±3.22a \| \| **Arginie** \| 2.46±0.13a \| 4.14±1.22a \| 0.50±0.03a \| 0.77±0.05ab \| 3.16±0.202a \| 4.76±0.3b \| 4.29±0.23a \| 6.32±0.35b \| 8.6±0.476a \| 11.8±0.65b \| \| **Ornithine** \| 0.86±0.04a \| 1.26±0.71b \| 0.04±0.003a \| 0.06±0.09a \| 0.15±0.011a \| 0.22±0.01a \| 0.16±0.01a \| 0.21±0.012a \| 0.91±0.051a \| 1.10±0.06a \| \| **Proline** \| 2.90±0.13a \| 4.14±1.31b \| 0.47±0.03a \| 0.63±0.03b \| 1.84±0.104a \| 2.27±0.12a \| 5.70±0.34a \| 9.05±0.54b \| 6.25±0.38a \| 9.88±0.59b \| \| **Asparagine** \| 3.56±0.21a \| 5.30±0.32b \| 0.82±0.04a \| 1.49±0.08b \| 1.256±0.07a \| 2.14±0.12b \| 24.79±1.5a \| 29.71±1.7ab \| 1.61±0.09a \| 1.91±0.11a \| \| **Isoleucine** \| 0.55±0.03a \| 0.71±0.04b \| 0.12±0.007a \| 0.16±0.01a \| 0.17±0.010a \| 0.36±0.02b \| 0.72±0.04a \| 1.01±0.06a \| 0.95±0.05a \| 1.31±0.07a \| \| **Leucine** \| 0.11±0.01a \| 0.16±0.01a \| 0.02±0.00a \| 0.026±0.00a \| 0.021±0.00a \| 0.031±0.0a \| 1.203±0.07a \| 1.52±0.088a \| 0.148±0.01a \| 0.19±0.01a \| \| **Methionine** \| 0.09±0.01a \| 0.14±0.01a \| 0.01±0.00a \| 0.023±0.01a \| 0.018±0.001a \| 0.027±0.00a \| 1.04±0.06a \| 1.50±0.08a \| 0.129±0.01a \| 0.17±0.01a \| \| **Threonine** \| 0.36±0.021a \| 0.41±0.02a \| 0.08±0.01a \| 0.15±0.00b \| 0.13±0.01a \| 0.16±0.01a \| 2.53±0.14a \| 3.03±0.17a \| 0.16±0.01a \| 0.26±0.15a \| \| **Valine** \| 2.54±0.22a \| 4.34±0.35b \| 1.32±0.14a \| 2.45±0.26a \| 1.06±0.11a \| 1.63±0.17a \| 5.17±0.42a \| 7.25±0.59b \| 18.69±1.52a \| 30.74±2.50b \| \| **Serine** \| 0.68±0.03a \| 0.87±0.05a \| 0.15±0.01a \| 0.19±0.01a \| 0.22±0.01a \| 0.44±0.03b \| 0.89±0.05a \| 1.24±0.07a \| 1.17±0.068a \| 1.76±0.80a \| \| **Phenylalanine** \| 3.93±0.25a \| 9.30±0.60b \| 0.43±0.037a \| 0.59±0.05a \| 0.22±0.019a \| 0.33±0.03a \| 1.13±0.07a \| 2.11±0.03b \| 5.98±0.39a \| 9.73±4.5b \| \| **Glycine** \| 0.55±0.04a \| 0.70±0.05a \| 1.16±0.11a \| 1.62±0.16a \| 0.84±0.084a \| 1.04±0.1a \| 5.29±0.41a \| 8.26±0.62b \| 5.20±0.39a \| 6.87±0.52a \| \| **Aspartaat** \| 0.18±0.01a \| 0.23±0.013a \| 0.03±0.002a \| 0.045±0.0a \| 0.033±0.002a \| 0.045±0.00a \| 0.17±0.010a \| 0.23±0.01a \| 0.17±0.01a \| 0.21±0.01a \| \| **Cystine** \| 3.34±0.19a \| 4.46±0.95a \| 0.02±0.00a \| 0.36±0.00b \| 0.032±0.002a \| 0.031±0.0a \| 0.08±0.01a \| 0.121±0.01a \| 0.00±0.00a \| 0.004±0.0a \| \| **Tyrosine** \| 3.51±0.13a \| 7.48±0.41b \| 0.52±0.03a \| 0.67±0.04a \| 0.44±0.029a \| 0.50±0.03a \| 3.54±0.19a \| 5.01±0.28b \| 4.89±0.27a \| 6.83±0.37b \|   **Supplementary Table 3.**   \|  \| **Ajwa C** \| **Ajwa T** \| **Hulwa C** \| **Hulwa T** \| **Ruthana C** \| **Ruthana T** \| **Sefri C** \| **Sefri T** \| **Luban C** \| **Luban T** \| \| --- \| --- \| --- \| --- \| --- \| --- \| --- \| --- \| --- \| --- \| --- \| \| **Oxalic acid** \| 1.44±0.05a \| 1.89±0.06a \| 1.68±0.51a \| 2.32±0.07a \| 1.45±0.05a \| 1.69±0.06a \| 1.64±0.05a \| 2.35±0.07b \| 1.27±0.04a \| 1.96±0.06a \| \| **Malic acid** \| 8.29±0.29a \| 11.40±0.40b \| 8.42±0.29a \| 11.52±0.42 \| 6.15±0.4a \| 8.13±0.53b \| 6.74±0.49a \| 9.61±0.70b \| 8.85±0.59a \| 15.2±1.03b \| \| **Succinic acid** \| 4.47±0.15a \| 6.49±0.2ab \| 1.32±0.04a \| 1.59±0.05a \| 4.98±0.3a \| 7.08±0.50b \| 0.88±0.16a \| 1.54±0.10b \| 0.66±0.04a \| 1.09±0.07b \| \| **Citric acid** \| 2.22±0.06a \| 3.22±0.09a \| 2.36±0.07a \| 3.011±0.10a \| 2.51±0.1a \| 3.20±0.24a \| 1.89±0.13a \| 2.56±0.17a \| 1.75±0.1a \| 2.9±0.18a \| \| **Isobutyric acid** \| 1.90±0.25a \| 2.76±0.08a \| 2.05±0.06a \| 2.63±0.078a \| 2.52±0.06a \| 2.67±0.08a \| 2.54±0.07a \| 3.37±0.90a \| 2.72±0.09a \| 3.60±0.00b \| \| **Furmaric acid** \| 0.28±0.00a \| 0.34±0.01a \| 0.25±0.01a \| 0.362±0.01b \| 0.32±0.01a \| 0.67±0.02b \| 0.18±0.01a \| 0.22±0.01a \| 0.30±0.01a \| 0.31±0.01a \|   **Supplementary Table 4.**   \|  \| **Ajwa C** \| **Ajwa T** \| **Hulwa C** \| **Hulwa T** \| **Ruthana C** \| **Ruthana T** \| **Sefri C** \| **Sefri T** \| **Luban C** \| **Luban T** \| \| --- \| --- \| --- \| --- \| --- \| --- \| --- \| --- \| --- \| --- \| --- \| \| **Dodecanoic (C12:0)** \| 0.29±0.003a \| 0.35±0.24a \| 0.39±0.00a \| 0.49±0.03a \| 0.34±0.00a \| 0.37±0.03a \| 0.36±0.00a \| 0.45±0.03a \| 0.49±0.02a \| 0.62±0.00b \| \| **Tetradecanoic (C14:0)** \| 0.07±0.001a \| 0.088±0.01a \| 0.08±0.00a \| 0.11±0.007a \| 0.09±0.001a \| 0.106±0.01a \| 0.119±0.01a \| 0.13±0.01a \| 0.09±0.00a \| 0.14±0.01b \| \| **Pentadecanoic (C15:0)** \| 0.01±0.00a \| 0.019±0.00a \| 0.017±0.00a \| 0.01±0.001a \| 0.017±0.00a \| 0.023±0.01a \| 0.021±0.00a \| 0.03±0.002a \| 0.02±0.00a \| 0.026±0.02a \| \| **Hexadecadienoic (C16:2)** \| 0.43±0.01a \| 0.56±0.04a \| 0.40±0.001a \| 0.48±0.032a \| 0.34±0.01a \| 0.45±0.03a \| 0.41±0.001a \| 0.52±0.036a \| 0.36±0.00a \| 0.55±0.04b \| \| **Heptadecanoic (C17:0)** \| 1.35±0.014a \| 1.90±0.13a \| 1.49±0.02a \| 1.72±0.116a \| 1.8±0.14a \| 1.43±0.09a \| 1.74±0.02a \| 2.24±0.01b \| 1.46±0.05a \| 2.0±0.14a \| \| **Octadecanoic (C18:0)** \| 0.03±0.001a \| 0.03±0.00a \| 0.02±0.001a \| 0.02±0.002a \| 0.022±0.0a \| 0.04±0.003a \| 0.02±0.00a \| 0.02±0.00a \| 0.02±0.00a \| 0.02±0.00a \| \| **Eicosanoic (C20:0)** \| 0.37±0.01a \| 0.56±0.04b \| 0.53±0.00a \| 0.69±0.00b \| 0.44±0.01a \| 0.60±0.04b \| 0.43±0.002a \| 0.59±0.04a \| 0.52±0.01a \| 0.69±0.04a \| \| **Docosanoic (C22:0)** \| 0.046±0.01a \| 0.06±0.00b \| 0.04±0.00a \| 0.05±0.00a \| 0.035±0.00a \| 0.05±0.00a \| 0.04±0.001a \| 0.05±0.00a \| 0.04±0.00a \| 0.04±0.00a \| \| **Tricosanoic (C23:0)** \| 0.01±0.00a \| 0.007±0.00a \| 0.004±0.00a \| 0.004±0.00a \| 0.004±0.00a \| 0.004±0.0a \| 0.003±0.00a \| 0.005±0.00a \| 0.003±0.00a \| 0.005±0.00a \| \| **Pentacosanoic (C25:0)** \| 0.15±0.00a \| 0.20±0.014a \| 0.095±0.00a \| 0.16±0.01b \| 0.129±0.01a \| 0.17±0.012a \| 0.10±0.001a \| 0.10±0.007a \| 0.14±0.00a \| 0.16±0.01a \| \| **Hexadecatrienoic (C16:3)** \| 0.11±0.003a \| 0.12±0.01a \| 0.08±0.003a \| 0.08±0.01a \| 0.1±0.00a \| 0.19±0.01b \| 0.13±0.001a \| 0.17±0.012a \| 0.16±0.00a \| 0.20±0.01a \| \| **Octadecenoic (18:1)** \| 0.03±0.00a \| 0.03±0.00a \| 0.02±0.001a \| 0.02±0.00a \| 0.025±0.0a \| 0.03±0.00a \| 0.035±0.00a \| 0.039±0.01a \| 0.04±0.01a \| 0.04±0.00a \| \| **Octadecatrienoic (C18:3)** \| 0.20±0.00a \| 0.39±0.00b \| 0.17±0.01a \| 0.21±0.02a \| 0.20±0.00a \| 0.27±0.02a \| 0.28±0.00a \| 0.38±0.0b \| 0.37±0.01a \| 0.42±0.02a \| \| **Eicosadienoic (C20:2)** \| 0.09±0.00a \| 0.09±0.01a \| 0.09±0.005a \| 0.12±0.01b \| 0.07±0.00a \| 0.07±0.01a \| 0.08±0.00a \| 0.090±0.01a \| 0.09±0.00a \| 0.12±0.01a \| \| **Tetracosenoic (C24:1)** \| 0.09±0.00a \| 0.15±0.01ab \| 0.06±0.00a \| 0.12±0.01b \| 0.082±0.01a \| 0.09±0.01a \| 0.07±0.00a \| 0.088±0.01a \| 0.07±0.00a \| 0.09±0.01b \| \| **Hexadecanoic (C16:0)** \| 2.40±0.23a \| 2.04±0.13a \| 1.47±0.013a \| 1.70±0.11a \| 1.28±0.02a \| 2.33±0.18a \| 1.97±0.12a \| 2.10±0.14a \| 7.86±2.09a \| 2.22±0.15b \| \| **Hexadecanoic (C16:1)** \| 0.08±0.00a \| 0.18±0.01b \| 0.07±0.001a \| 0.08±0.01a \| 0.07±0.0a \| 0.10±0.01a \| 0.064±0.01a \| 0.104±0.01a \| 0.058±0.00a \| 1.52±0.48b \| \| **Octadecadienoic (C18:2)** \| 0.01±0.00a \| 0.01±0.00a \| 0.011±0.00a \| 0.014±0.0a \| 0.013±0.0a \| 0.01±0.00a \| 0.011±0.00a \| 0.016±0.00a \| 0.01±0.00a \| 0.012±0.00a \| \| **Tetracosanoic (C24:0)** \| 0.05±0.00a \| 0.07±0.00b \| 0.36±0.10a \| 0.06±0.00b \| 0.033±0.0a \| 0.05±0.01a \| 0.05±0.00a \| 0.07±0.00b \| 0.05±0.001a \| 0.06±0.004a \| \| **Hexacosanoic (26:0)** \| 0.003±0.00a \| 0.002±0.01a \| 0.002±0.00a \| 0.18±0.00b \| 0.002±0.0a \| 0.002±0.00a \| 0.002±0.00a \| 0.002±0.00a \| 0.002±0.00a \| 0.003±0.00a \|   **Supplementary Table 5.**   \|  \| **Ajwa C** \| **Ajwa T** \| **Hulwa C** \| **Hulwa T** \| **Ruthana C** \| **Ruthana T** \| **Sefri C** \| **Sefri T** \| **Luban C** \| **Luban T** \| \| --- \| --- \| --- \| --- \| --- \| --- \| --- \| --- \| --- \| --- \| --- \| \| **β-glucogallin** \| 2.31±0.16a \| 4.7±0.03b \| 1.8±0.19a \| 2.6±0.10a \| 2.07±0.3a \| 2.69±0.25a \| 2.33±0.12a \| 3.59±0.1a \| 1.94±0.03a \| 3.12±0.00b \| \| **Caffeic acid** \| 0.03±0.00a \| 0.03±0.00a \| 0.016±0.01a \| 0.02±0.0a \| 0.02±0.0a \| 0.02±0.04a \| 0.024±0.0a \| 0.03±0.0a \| 0.033±0.0a \| 0.04±0.00a \| \| **Ferulic acid** \| 0.07±0.01a \| 0.08±0.01a \| 0.04±0.01a \| 0.05±0.01a \| 0.06±0.01a \| 0.07±0.01a \| 0.103±0.01a \| 0.11±0.02a \| 0.51±0.01a \| 0.57±0.01a \| \| **Protocatechuic acid** \| 0.22±0.02a \| 0.24±0.03a \| 0.14±0.03a \| 0.15±0.04a \| 0.19±0.02a \| 0.21±0.03a \| 0.31±0.05a \| 0.34±0.06a \| 1.53±0.02a \| 1.73±0.03a \| \| **Catechin** \| 0.82±0.12a \| 0.91±0.10a \| 0.51±0.12a \| 0.57±0.14a \| 0.74±0.09a \| 0.78±0.14a \| 0.79±0.03a \| 0.89±0.04a \| 0.66±0.01a \| 0.73±0.01a \| \| **Galic acid** \| 7.32±0.85a \| 8.1±0.93a \| 4.55±1.11a \| 4.98±1.22a \| 6.23±0.86a \| 6.78±0.94a \| 6.99±0.31a \| 7.50±0.19a \| 5.86±0.92a \| 6.42±0.11a \| \| **p-Coumaric acid** \| 4.72±0.54a \| 5.19±0.60a \| 2.9±0.71a \| 3.16±0.77a \| 4.02±0.55a \| 4.44±0.61a \| 4.51±0.20a \| 4.9±0.22a \| 3.78±0.06a \| 4.12±0.66a \| \| **Resorcinol** \| 0.04±0.01a \| 0.12±0.04b \| 0.03±0.01a \| 0.03±0.01a \| 0.04±0.01a \| 0.04±0.01a \| 0.05±0.00a \| 0.05±0.00a \| 0.03±0.00a \| 0.04±0.001a \| \| **Chlorogenic acid** \| 0.28±0.04a \| 0.32±0.04a \| 0.18±0.04a \| 0.19±0.048a \| 0.24±0.03a \| 0.26±0.03a \| 0.27±0.01a \| 0.3±0.014a \| 0.23±0.01a \| 0.25±0.01a \| \| **Syringic acid** \| 1.29±0.15a \| 1.43±0.16a \| 0.85±0.19a \| 0.90±0.22a \| 1.14±0.15a \| 1.21±0.17a \| 1.24±0.05a \| 1.35±0.6a \| 1.03±0.01a \| 1.16±0.02a \| \| **Quercetin** \| 1.94±0.22a \| 3.3±0.383b \| 1.21±0.37a \| 2.05±0.50b \| 1.65±0.2a \| 3.05±0.04b \| 1.86±0.08a \| 3.3±0.15b \| 1.5±0.02a \| 2.6±0.04b \| \| **Quercetrin** \| 0.21±0.02a \| 0.23±0.03a \| 0.13±0.03a \| 0.25±0.00b \| 0.18±0.02a \| 0.24±0.02a \| 0.21±0.01a \| 0.24±0.01a \| 0.17±0.0a \| 0.19±0.00a \| \| **Luteolin** \| 0.06±0.01a \| 0.11±0.01a \| 0.04±0.01a \| 0.06±0.00b \| 0.06±0.01a \| 0.09±0.01a \| 0.06±0.00a \| 0.10±0.01a \| 0.052±0.0a \| 0.09±0.00a \| \| **Apigenin** \| 0.41±0.05a \| 0.71±0.08b \| 0.26±0.06a \| 0.44±0.10a \| 0.35±0.05a \| 0.62±0.09a \| 0.40±0.02a \| 0.67±0.30a \| 0.33±0.0a \| 0.54±0.01a \| \| **Isoquercetrin** \| 1.04±0.12a \| 1.60±0.18a \| 0.65±0.16a \| 1.07±0.00b \| 0.89±0.12a \| 1.64±0.23b \| 1.04±0.04a \| 1.61±0.07a \| 0.5±0.01a \| 0.83±0.013a \| \| **Rutin** \| 1.36±0.15a \| 2.40±0.28b \| 0.84±0.25a \| 1.4±0.34b \| 1.16±0.16a \| 2.16±0.30a \| 1.3±0.058a \| 2.2±0.09a \| 1.09±0.02a \| 1.80±0.00ab \| \| **Ellagic acid** \| 0.31±0.02a \| 0.64±0.05a \| 0.19±0.04a \| 0.31±0.07b \| 0.26±0.03a \| 0.49±0.05b \| 0.30±0.02a \| 0.55±0.03a \| 0.32±0.01a \| 0.52±0.02a \| \| **Velutin** \| 0.36±0.03a \| 0.63±0.06b \| 0.23±0.05a \| 0.37±0.08a \| 0.00±0.0a \| 0.00±0.00a \| 0.36±0.02a \| 0.60±0.04a \| 0.43±0.01a \| 0.71±0.02a \| \| **Naringenin** \| 0.005±0.00a \| 0.009±0.0b \| 0.003±0.00a \| 0.005±0.07a \| 0.004±0.00a \| 0.01±0.00a \| 0.005±0.0a \| 0.01±0.0b \| 0.00±0.001a \| 0.00±0.00a \| \| **Genistein** \| 0.00±0.00a \| 0.00±0.00a \| 0.01±0.00a \| 0.003±0.05a \| 0.003±0.00a \| 0.004±0.00a \| 0.003±0.0a \| 0.005±0.0a \| 0.00±0.001a \| 0.00±0.001a \| \| **Daidzein** \| 0.002±0.00a \| 0.003±0.00a \| 0.00±0.002a \| 0.00±0.002a \| 0.001±0.00a \| 0.002±0.00a \| 0.00±0.00a \| 0.00±0.0a \| 0.003±0.00a \| 0.00±0.00a \| \| **Fisetin** \| 0.002±0.00a \| 0.004±0.0b \| 0.00±0.00a \| 0.00±0.001a \| 0.002±0.00a \| 0.003±0.00a \| 0.002±0.00a \| 0.003±0.0a \| 0.00±0.000a \| 0.003±0.00a \| \| **O-hydroxydaidzein** \| 0.003±0.00a \| 0.005±0.0b \| 0.002±0.00a \| 0.003±0.0a \| 0.00±0.00a \| 0.00±0.00a \| 0.003±0.0a \| 0.004±0.0a \| 0.002±0.00a \| 0.004±0.00a \|   **Supplementary Table 6.**   \|  \| **Ajwa C** \| **Ajwa T** \| **Hulwa C** \| **Hulwa T** \| **Ruthana C** \| **Ruthana T** \| **Sefri C** \| **Sefri T** \| **Luban C** \| **Luban T** \| \| --- \| --- \| --- \| --- \| --- \| --- \| --- \| --- \| --- \| --- \| --- \| \| **Alfa toc.** \| 0.14±0.01a \| 0.17±0.01a \| 0.25±0.01a \| 0.22±0.03a \| 0.09±0.00a \| 0.14±0.01b \| 0.2±0.01a \| 0.22±0.00a \| 0.2±0.01a \| 0.23±0.00a \| \| **Betha toc.** \| 0.03±0.00a \| 0.15±0.06b \| 0.02±0.0a \| 0.06±0.00b \| 0.02±0.12a \| 0.06±0.02b \| 0.03±0.22a \| 0.07±0.02b \| 0.03±0.3a \| 0.06±0.01b \| \| **Gamma toc.** \| 0.02±0.00a \| 0.12±0.05b \| 0.03±0.0a \| 0.05±0.00b \| 0.02±0.02a \| 0.05±0.01b \| 0.05±0.00a \| 0.07±0.01a \| 0.02±0.00a \| 0.05±0.01b \| \| **Delta toc.** \| 0.01±0.00a \| 0.02±0.00a \| 0.02±0.02a \| 0.07±0.00b \| 0.01±0.02a \| 0.01±0.00a \| 0.04±0.01a \| 0.05±0.01a \| 0.01±0.00a \| 0.06±0.02b \| \| **Total tocopherol** \| 0.24±0.01a \| 0.49±0.10b \| 0.31±0.02a \| 0.57±0.14a \| 0.14±0.00a \| 0.29±0.07b \| 0.27±0.01a \| 0.26±0.03a \| 0.26±0.01a \| 0.45±0.08b \| \| **α-Carotene (Vit-A)** \| 0.44±0.02a \| 0.43±0.05a \| 0.47±0.02a \| 0.41±0.11a \| 0.3±0.01a \| 0.34±0.04a \| 0.4±0.022a \| 0.4±0.04a \| 0.51±0.02a \| 0.49±0.06b \| \| **β-Carotene (Vit-A)** \| 0.36±0.01a \| 0.48±0.02a \| 0.29±0.02a \| 0.39±0.04a \| 0.48±0.03a \| 0.44±0.06a \| 0.27±0.01a \| 0.82±0.20b \| 0.48±0.03a \| 1.16±0.09b \| \| **β-Cryptoxanthin (Vit-A)** \| 0.29±0.01a \| 0.61±0.01b \| 0.02±0.01a \| 0.05±0.00b \| 0.67±0.05a \| 0.75±0.03a \| 0.27±0.08a \| 0.75±0.34b \| 0.4±0.02a \| 0.64±0.14a \| \| **Thiamine (Vit-B)** \| 0.32±0.01a \| 0.26±0.06a \| 0.35±0.01a \| 0.27±0.06a \| 0.67±0.03a \| 0.54±0.10a \| 0.22±0.01a \| 0.19±0.04a \| 0.26±0.01a \| 0.21±0.01a \| \| **Phylloquinone (Vit-K)** \| 0.11±0.00a \| 0.09±0.02a \| 0.46±0.00a \| 0.79±0.00b \| 0.29±0.01a \| 0.28±0.06a \| 0.38±0.01a \| 0.45±0.01a \| 0.25±0.01a \| 0.25±0.01a \|   **Supplementary Table 7.**   \|  \| **Ajwa C** \| **Ajwa T** \| **Hulwa C** \| **Hulwa T** \| **Ruthana C** \| **Ruthana T** \| **Sefri C** \| **Sefri T** \| **Luban C** \| **Luban T** \| \| --- \| --- \| --- \| --- \| --- \| --- \| --- \| --- \| --- \| --- \| --- \| \| **K** \| 4.9±0.06a \| 12.04±0.71b \| 2.44±0.03a \| 11.14±4.6b \| 4.14±0.05a \| 11.91±0.41b \| 1.12±0.01a \| 9.81±4.8b \| 3.00±0.03a \| 12.0±4.71b \| \| **Ca** \| 0.21±0.01a \| 2.21±1.10b \| 0.12±0.00a \| 2.64±1.5b \| 0.20±0.0a \| 2.63±1.24b \| 0.43±0.00a \| 1.73±0.9b \| 0.12±0.0a \| 1.82±1.01b \| \| **Mg** \| 0.60±0.01a \| 1.91±0.6b \| 0.31±0.01a \| 1.9±0.9b \| 0.51±0.01a \| 1.23±0.31b \| 0.13±0.00a \| 1.00±0.50b \| 0.38±0.01a \| 1.54±0.68a \| \| **P** \| 3.9±0.12a \| 7.91±1.4b \| 4.62±0.2a \| 2.34±0.9b \| 3.0±0.2a \| 2.35±0.74a \| 4.74±0.08a \| 5.20±0.21a \| 2.72±0.21a \| 7.60±0.34b \| \| **Na** \| 0.06±0.00a \| 0.52±0.2b \| 0.03±0.00a \| 0.06±0.01b \| 0.05±0.0a \| 0.39±0.22b \| 0.04±0.00a \| 0.75±0.41b \| 0.05±0.01a \| 0.47±0.20b \| \| **Cu** \| 0.09±0.00a \| 0.36±0.9a \| 0.07±0.00a \| 0.62±0.3b \| 0.08±0.0a \| 0.83±0.40b \| 0.49±0.01a \| 0.95±0.44a \| 0.04±0.00a \| 0.64±0.40a \| \| **Fe** \| 0.002±0.00a \| 0.21±0.0b \| 0.00±0.00a \| 0.11±0.00b \| 0.002±0.0a \| 0.48±0.00b \| 0.01±0.00a \| 0.85±0.01b \| 0.001±0.0a \| 0.24±0.10a \| \| **Mn** \| 0.05±0.00a \| 0.12±0.0b \| 0.03±0.00a \| 0.04±0.0a \| 0.05±0.0a \| 0.07±0.00a \| 0.04±0.00a \| 0.08±0.00b \| 0.05±0.00a \| 0.08±0.00b \| \| **Cd** \| 0.00±0.00a \| 0.02±0.0b \| 0.00±0.00a \| 0.02±0.1a \| 0.002±0.0a \| 0.012±0.0b \| 0.00±0.00a \| 0.004±0.00a \| 0.00±0.00a \| 0.02±0.00a \| \| **Zn** \| 0.01±0.00a \| 0.05±0.02a \| 0.01±0.00a \| 0.51±0.2b \| 0.01±0.0a \| 0.06±0.02b \| 0.01±0.00a \| 0.14±0.07ab \| 0.01±0.00a \| 0.49±0.20b \| \| **N** \| 3.82±0.02a \| 5.44±0.05b \| 5.12±0.03a \| 4.62±0.14a \| 2.72±0.09a \| 6.55±0.20b \| 6.53±0.20a \| 5.51±0.06ab \| 3.44±0.05a \| 8.71±0.07b \|   **Supplementary Table 8.**   \|  \| \| **Ajwa C** \| \| **Ajwa T** \| \| **Hulwa C** \| \| **Hulwa T** \| \| **Ruthana C** \| \| **Ruthana T** \| \| **Sefri C** \| \| **Sefri T** \| **Luban C** \| **Luban T** \| \| \| --- \| --- \| --- \| --- \| --- \| --- \| --- \| --- \| --- \| --- \| --- \| --- \| --- \| --- \| --- \| --- \| --- \| --- \| --- \| --- \| \| **TAC (FRAP)** \| 6.18±0.03a \| \| 9.07±0.06b \| \| 6.46±0.61a \| \| 10.34±0.4b \| \| 56.82±0.7a \| \| 102.34±2.1b \| \| 61.34±1.11a \| \| 109.6±1.5b \| \| 65.85±0.87a \| \| 117.12±2.1b \| \| \| **TAC (DPPH %)** \| 28.51±0.10a \| \| 39.11±0.3b \| \| 33.20±3.02a \| \| 53.17±3.35b \| \| 76.31±1a \| \| 125.11±1.8b \| \| 36.4±0.61a \| \| 151.9±2.9b \| \| 88.8±0.24a \| \| 178.62±3.0b \| \| \| **Antiprotozoal**  ***(Trypanosoma cruzi* )** \| 33.19±2.01a \| \| 50.94±.6b \| \| 31.46±3.07a \| \| 49.50±4.39b \| \| 6.18±0.1a \| \| 10.1±0.2ab \| \| 4.37±0.12a \| \| 10.20±0.2b \| \| 2.6±0.06a \| \| 10.20±0.25b \| \| \| **Anti-bacterial**  **(*Streptococcus spp.*)** \| 9.50±0.24a \| \| 16.51±0.1b \| \| 18.80±3.04a \| \| 23.3±1.11a \| \| 30.06±0.4a \| \| 48.45±1.01b \| \| 23.9±0.42a \| \| 47.6±2.41b \| \| 17.81±0.44a \| \| 46.80±2.61b \| \| \| **Anti-bacterial**  **(*Escherichia coli)*** \| 28.00±0.90a \| \| 36.91±0.8b \| \| 27.26±1.71a \| \| 38.61±1.12b \| \| 31.76±0.6a \| \| 52.84±0.94b \| \| 19.76±0.40a \| \| 54.20±0.74b \| \| 37.72±0.12a \| \| 55.71±1.44b \| \| \| **Anti-Cancer (HepG2)** \| 72.94±0.89a \| \| 112.31±3b \| \| 86.81±6.01a \| \| 87.09±1a \| \| 96.00±1.8a \| \| 122.85±4.7b \| \| 109.12±2.4a \| \| 126.32±2.1a \| \| 122.0±2.41a \| \| 129.8±2.01a \| \| \| **Anti-Cancer (Colo205)** \| 101.91±5.51a \| \| 123.2±2.01b \| \| 114±13.01a \| \| 109.21±1.2a \| \| 98.00±2a \| \| 111.92±9.2a \| \| 110.88±1-9a \| \| 147.52±11ab \| \| 123.65±1.8a \| \| 183.15±2.3b \| \| \| **Anti-Cancer (293)** \| 113.47±0.64a \| \| 120.10±1b \| \| 108.7±16.7a \| \| 96.39±1.65a \| \| 114.41±2a \| \| 129.32±3.0b \| \| 111.92±2.0a \| \| 134.71±2.4b \| \| 109.40±43.1a \| \| 140.15±17.1b \| \| |  |  |  |  |  |
| --- | --- | --- | --- | --- | --- | --- | --- | --- | --- | --- | --- | --- | --- | --- | --- | --- | --- | --- | --- | --- | --- | --- | --- | --- | --- | --- | --- | --- | --- | --- | --- | --- | --- | --- | --- | --- | --- | --- | --- | --- | --- | --- | --- | --- | --- | --- | --- | --- | --- | --- | --- | --- | --- | --- | --- | --- | --- | --- | --- | --- | --- | --- | --- | --- | --- | --- | --- | --- | --- | --- | --- | --- | --- | --- | --- | --- | --- | --- | --- | --- | --- | --- | --- | --- | --- | --- | --- | --- | --- | --- | --- | --- | --- | --- | --- | --- | --- | --- | --- | --- | --- | --- | --- | --- | --- | --- | --- | --- | --- | --- | --- | --- | --- | --- | --- | --- | --- | --- | --- | --- | --- | --- | --- | --- | --- | --- | --- | --- | --- | --- | --- | --- | --- | --- | --- | --- | --- | --- | --- | --- | --- | --- | --- | --- | --- | --- | --- | --- | --- | --- | --- | --- | --- | --- | --- | --- | --- | --- | --- | --- | --- | --- | --- | --- | --- | --- | --- | --- | --- | --- | --- | --- | --- | --- | --- | --- | --- | --- | --- | --- | --- | --- | --- | --- | --- | --- | --- | --- | --- | --- | --- | --- | --- | --- | --- | --- | --- | --- | --- | --- | --- | --- | --- | --- | --- | --- | --- | --- | --- | --- | --- | --- | --- | --- | --- | --- | --- | --- | --- | --- | --- | --- | --- | --- | --- | --- | --- | --- | --- | --- | --- | --- | --- | --- | --- | --- | --- | --- | --- | --- | --- | --- | --- | --- | --- | --- | --- | --- | --- | --- | --- | --- | --- | --- | --- | --- | --- | --- | --- | --- | --- | --- | --- | --- | --- | --- | --- | --- | --- | --- | --- | --- | --- | --- | --- | --- | --- | --- | --- | --- | --- | --- | --- | --- | --- | --- | --- | --- | --- | --- | --- | --- | --- | --- | --- | --- | --- | --- | --- | --- | --- | --- | --- | --- | --- | --- | --- | --- | --- | --- | --- | --- | --- | --- | --- | --- | --- | --- | --- | --- | --- | --- | --- | --- | --- | --- | --- | --- | --- | --- | --- | --- | --- | --- | --- | --- | --- | --- | --- | --- | --- | --- | --- | --- | --- | --- | --- | --- | --- | --- | --- | --- | --- | --- | --- | --- | --- | --- | --- | --- | --- | --- | --- | --- | --- | --- | --- | --- | --- | --- | --- | --- | --- | --- | --- | --- | --- | --- | --- | --- | --- | --- | --- | --- | --- | --- | --- | --- | --- | --- | --- | --- | --- | --- | --- | --- | --- | --- | --- | --- | --- | --- | --- | --- | --- | --- | --- | --- | --- | --- | --- | --- | --- | --- | --- | --- | --- | --- | --- | --- | --- | --- | --- | --- | --- | --- | --- | --- | --- | --- | --- | --- | --- | --- | --- | --- | --- | --- | --- | --- | --- | --- | --- | --- | --- | --- | --- | --- | --- | --- | --- | --- | --- | --- | --- | --- | --- | --- | --- | --- | --- | --- | --- | --- | --- | --- | --- | --- | --- | --- | --- | --- | --- | --- | --- | --- | --- | --- | --- | --- | --- | --- | --- | --- | --- | --- | --- | --- | --- | --- | --- | --- | --- | --- | --- | --- | --- | --- | --- | --- | --- | --- | --- | --- | --- | --- | --- | --- | --- | --- | --- | --- | --- | --- | --- | --- | --- | --- | --- | --- | --- | --- | --- | --- | --- | --- | --- | --- | --- | --- | --- | --- | --- | --- | --- | --- | --- | --- | --- | --- | --- | --- | --- | --- | --- | --- | --- | --- | --- | --- | --- | --- | --- | --- | --- | --- | --- | --- | --- | --- | --- | --- | --- | --- | --- | --- | --- | --- | --- | --- | --- | --- | --- | --- | --- | --- | --- | --- | --- | --- | --- | --- | --- | --- | --- | --- | --- | --- | --- | --- | --- | --- | --- | --- | --- | --- | --- | --- | --- | --- | --- | --- | --- | --- | --- | --- | --- | --- | --- | --- | --- | --- | --- | --- | --- | --- | --- | --- | --- | --- | --- | --- | --- | --- | --- | --- | --- | --- | --- | --- | --- | --- | --- | --- | --- | --- | --- | --- | --- | --- | --- | --- | --- | --- | --- | --- | --- | --- | --- | --- | --- | --- | --- | --- | --- | --- | --- | --- | --- | --- | --- | --- | --- | --- | --- | --- | --- | --- | --- | --- | --- | --- | --- | --- | --- | --- | --- | --- | --- | --- | --- | --- | --- | --- | --- | --- | --- | --- | --- | --- | --- | --- | --- | --- | --- | --- | --- | --- | --- | --- | --- | --- | --- | --- | --- | --- | --- | --- | --- | --- | --- | --- | --- | --- | --- | --- | --- | --- | --- | --- | --- | --- | --- | --- | --- | --- | --- | --- | --- | --- | --- | --- | --- | --- | --- | --- | --- | --- | --- | --- | --- | --- | --- | --- | --- | --- | --- | --- | --- | --- | --- | --- | --- | --- | --- | --- | --- | --- | --- | --- | --- | --- | --- | --- | --- | --- | --- | --- | --- | --- | --- | --- | --- | --- | --- | --- | --- | --- | --- | --- | --- | --- | --- | --- | --- | --- | --- | --- | --- | --- | --- | --- | --- | --- | --- | --- | --- | --- | --- | --- | --- | --- | --- | --- | --- | --- | --- | --- | --- | --- | --- | --- | --- | --- | --- | --- | --- | --- | --- | --- | --- | --- | --- | --- | --- | --- | --- | --- | --- | --- | --- | --- | --- | --- | --- | --- | --- | --- | --- | --- | --- | --- | --- | --- | --- | --- | --- | --- | --- | --- | --- | --- | --- | --- | --- | --- | --- | --- | --- | --- | --- | --- | --- | --- | --- | --- | --- | --- | --- | --- | --- | --- | --- | --- | --- | --- | --- | --- | --- | --- | --- | --- | --- | --- | --- | --- | --- | --- | --- | --- | --- | --- | --- | --- | --- | --- | --- | --- | --- | --- | --- | --- | --- | --- | --- | --- | --- | --- | --- | --- | --- | --- | --- | --- | --- | --- | --- | --- | --- | --- | --- | --- | --- | --- | --- | --- | --- | --- | --- | --- | --- | --- | --- | --- | --- | --- | --- | --- | --- | --- | --- | --- | --- | --- | --- | --- | --- | --- | --- | --- | --- | --- | --- | --- | --- | --- | --- | --- | --- | --- | --- | --- | --- | --- | --- | --- | --- | --- | --- | --- | --- | --- | --- | --- | --- | --- | --- | --- | --- | --- | --- | --- | --- | --- | --- | --- | --- | --- | --- | --- | --- | --- | --- | --- | --- | --- | --- | --- | --- | --- | --- | --- | --- | --- | --- | --- | --- | --- | --- | --- | --- | --- | --- | --- | --- | --- | --- | --- | --- | --- | --- | --- | --- | --- | --- | --- | --- | --- | --- | --- | --- | --- | --- | --- | --- | --- | --- | --- | --- | --- | --- | --- | --- | --- | --- | --- | --- | --- | --- | --- | --- | --- | --- | --- | --- | --- | --- | --- | --- | --- | --- | --- | --- | --- | --- | --- | --- | --- | --- | --- | --- | --- | --- | --- | --- | --- | --- | --- | --- | --- | --- | --- | --- | --- | --- | --- | --- | --- | --- | --- | --- | --- | --- | --- | --- | --- | --- | --- | --- | --- | --- | --- | --- | --- | --- | --- | --- | --- | --- | --- | --- | --- | --- | --- | --- | --- | --- | --- | --- | --- | --- | --- | --- | --- | --- | --- | --- | --- | --- | --- | --- | --- | --- | --- | --- | --- | --- | --- | --- | --- | --- | --- | --- | --- | --- | --- | --- | --- | --- | --- | --- | --- | --- | --- | --- | --- | --- | --- | --- | --- | --- | --- | --- | --- | --- | --- | --- | --- | --- | --- | --- | --- | --- | --- | --- | --- | --- | --- | --- | --- | --- | --- | --- | --- | --- | --- | --- | --- | --- | --- | --- | --- | --- | --- | --- | --- | --- | --- | --- | --- | --- | --- | --- | --- | --- | --- | --- | --- | --- | --- | --- | --- | --- | --- | --- | --- | --- | --- | --- | --- | --- | --- | --- | --- | --- | --- | --- | --- | --- | --- | --- | --- | --- | --- | --- | --- | --- | --- | --- | --- | --- | --- | --- | --- | --- | --- | --- | --- | --- | --- | --- | --- | --- | --- | --- | --- | --- | --- | --- | --- | --- | --- | --- | --- | --- | --- | --- | --- | --- | --- | --- | --- | --- | --- | --- | --- | --- | --- | --- | --- | --- | --- | --- | --- | --- | --- | --- | --- | --- | --- | --- | --- | --- | --- | --- | --- | --- | --- | --- | --- | --- | --- | --- | --- | --- | --- | --- | --- | --- | --- | --- | --- | --- | --- | --- | --- | --- | --- | --- | --- | --- | --- | --- | --- | --- | --- | --- | --- | --- | --- | --- |

**Supplementary Table 9.**

| Microorganisms | DD ^a^ | MIC | MBC |
| --- | --- | --- | --- |
| Gram Positive Bacteria |  |  |  |
| Streptococcus sp | 16 | 250 | 250 |
| Serratia marcescens | 23 | - | - |
| Staphylococcus aureus | 22 | 125 | 125 |
| Gram Negative Bacteria |  |  |  |
| Escherichia coli | 15 | 250 | 250 |
| Klebsiella pneumoniae | 12 | 250 | 250 |
| Proteus vulgaris | 16 | - | - |
| Pseudomonas aeruginosa | 17 | - | - |
| Yeast |  |  |  |
| Candida albicans | 13 | 500 | 500 |

MIC (minimum inhibition concentration) and MBC (minimum bactericidal concentration) as μg/ml of methanol extract; (−) no antimicrobial activity. Values are average of triplicate. ^a^ Inhibition zone in diameter (mm) around the discs impregnated with methanol extract (100 μg/disc)

**Supplementary Table 10.**

| **Test items** | **Concentration (μg /ml)** |  | **Inhibition % of Revertants** | | |
| --- | --- | --- | --- | --- | --- |
|  |  |  | **TA98** |  | **TA100** |
| **Positive control** |  |  |  |  |  |
| NPD* | **3** |  | **-** |  | **-** |
| NaN_3_* | **8** |  | **-** |  | **-** |
| **β-D-glucogalline** | **2500** |  | **45** |  | **36** |
|  | **1500** |  | **39** |  | **20** |
|  | **500** |  | **35** |  | **18** |
|  | **250** |  | **29** |  | **12** |

- 4-NPD and NaN3 were used as positive controls for S. typhimurium TA98 and TA100 strains, respectively.
- Data shown are Mean±SD of experiments with triplicate plates/concentration/experiment.

**Supplementary Table 11.**

| *Test organism* | Standard (Benznidazole, Miltefosine) | β-D-glucogalline |
| --- | --- | --- |
| *T. cruzi* | 0.056±0.00^a^ | 4.84±1.50^b^ |
| *L. donovani* | 0.301±0.03^a^ | 8.63±5.87^b^ |

**Supplementary Table 12.**

| *Anrtioxidant test* | Standard (Gallic acid) | **β-D-glucogalline** |
| --- | --- | --- |
| DPPH (%) | 34.86±4.21 ^a^ | 49.25±5.32^b^ |
| FRAP (nmol /gFW) | 31.34±2.62^a^ | 39.44±2.20c |

**Supplementary Figure 1.**


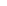

Supplement: FIGURE S1 — Effect of different concentrations of β-D-glucogallin, 0 (control, ◆), 100 μg/ml (■), 200 μg/ml (▲) on the viability of Staphylococcus aureus. Values are the average of three individual replicates (means ± SD). Different letters represent significant differences between the treatments (Duncan test; P < 0.05 and n = 4). [file Data_Sheet_1.docx]
